# Exploring the rice dispensable genome using a metagenome-like assembly strategy

**DOI:** 10.1186/s13059-015-0757-3

**Published:** 2015-09-07

**Authors:** Wen Yao, Guangwei Li, Hu Zhao, Gongwei Wang, Xingming Lian, Weibo Xie

**Affiliations:** National Key Laboratory of Crop Genetic Improvement, National Center of Plant Gene Research, Huazhong Agricultural University, Wuhan, 430070 China; Agricultural Bioinformatics Key Laboratory of Hubei Province, College of Informatics, Huazhong Agricultural University, Wuhan, 430070 China

## Abstract

**Background:**

The dispensable genome of a species, consisting of the dispensable sequences present only in a subset of individuals, is believed to play important roles in phenotypic variation and genome evolution. However, construction of the dispensable genome is costly and labor-intensive at present, and so the influence of the dispensable genome in genetic and functional genomic studies has not been fully explored.

**Results:**

We construct the dispensable genome of rice through a metagenome-like de novo assembly strategy based on low-coverage (1–3×) sequencing data of 1483 cultivated rice (*Oryza sativa* L.) accessions. Thousands of protein-coding genes are successfully assembled, including most of the known agronomically important genes absent from the Nipponbare rice reference genome. We develop an integration approach based on alignment and linkage disequilibrium, which is able to assign genomic positions relative to the reference genome for more than 78.2 % of the dispensable sequences. We carry out association mapping studies for rice grain width and 840 metabolic traits using 0.46 million polymorphisms between the dispensable sequences of different rice accessions. About 23.5 % of metabolic traits have more significant association signals with polymorphisms from dispensable sequences than with SNPs from the reference genome, and 41.6 % of trait-associated SNPs have concordant genomic locations with associated dispensable sequences.

**Conclusions:**

Our results suggest the feasibility of building a species’ dispensable genome using low-coverage population sequencing data. The constructed sequences will be helpful for understanding the rice dispensable genome and are complementary to the reference genome for identifying candidate genes associated with phenotypic variation.

**Electronic supplementary material:**

The online version of this article (doi:10.1186/s13059-015-0757-3) contains supplementary material, which is available to authorized users.

## Background

The pan-genome concept refers to the non-redundant collection of all DNA sequences present in the entire population of a species [1, 2], which comprises a “core genome” containing sequences present in all individuals, a “dispensable genome” containing sequences present in two or more individuals [3], and unique sequences specific to an individual [4]. Previous studies showed the existence of individual-specific and population-specific DNA sequences in different organisms [1, 5]. Traditional studies tended to use several finished genome sequences to discover dispensable sequences and build a species’ pan-genome [6, 7]. However, decoding the complete genome of plants or animals is still labor-intensive and costly at present. Alternatively, a recent study characterized the maize pan-transcriptome using sequencing of RNAs from different maize inbred lines [5]. They obtained tens of thousands of dispensable sequences by assembling sequencing data for each line separately. However, such a method could not discover genes expressed at low levels or in specific tissues and the obtained sequences were usually very short [5].

With the application of next-generation sequencing, huge amounts of low-coverage population sequencing data have been generated [8, 9]. These data were usually generated for genome-wide association studies (GWASs) and population genomic studies, which mainly focused on the single nucleotide polymorphisms (SNPs) or insertions or deletions (INDELs) in reference genomic regions [8, 10]. A large portion of individual- and subpopulation-specific sequences were left out and present studies have not taken full advantage of the huge amount of population sequencing data.

Rice (*Oryza sativa* L.) is a staple food crop and an ideal model for functional genomic research of monocots. The availability of the high quality Nipponbare rice reference genome sequence has greatly accelerated gene cloning [11]; over 600 genes were cloned by the end of 2010 [12]. However, a lot of genes controlling important traits have been found to be absent from the Nipponbare reference genome [11], such as *GW5* [13], *Sub1A* [14], and *Pikm-1* [15]. This indicates that one genome is insufficient [2]. More genome sequences are needed to gain a more comprehensive understanding of the pan-genome of rice.

The absence of important genes from the rice reference genome, and the availability of population resequencing data [8, 9], prompted us to develop methods to identify the sequences that were absent from the reference genome by de novo assembly of population sequencing data. These sequences are important components of the rice dispensable genome.

In this study, we show the feasibility of using only the population sequencing data with each sample sequenced at very low coverage (1–3×) to identify the DNA sequences present in some or all of these cultivated rice accessions but absent from the Nipponbare reference genome. A metagenome-like assembly strategy was adopted to assemble the reads of various rice accessions to build the sequence map of the rice dispensable genome. We further demonstrate that these sequences would be helpful for understanding the rice dispensable genome and picking candidate genes in quantitative trait locus (QTL) mapping and GWASs in rice. The assembled sequences and other information have been deposited in the Panrice database [16]. Users may query this database using accession number or DNA/protein sequence.

## Results

### Collecting sequence data and assembling the dispensable genome using a metagenome-like assembly strategy

We collected data of 533 rice accessions sequenced at ~2.5× coverage and 950 rice accessions sequenced at ~1× coverage (based on a 384-Mb genome size of the Nipponbare reference genome; Additional file 1) [8, 9, 17–19]. The two data sets consist of low-coverage sequencing data of 1483 cultivated rice accessions from 73 countries, comprising 11.3 billion reads. These reads were mapped to the Nipponbare genome (release 6.1 of the Michigan State University (MSU) Rice Genome Annotation Project) using BWA [20], providing ~2400-fold coverage of the genome. The details of the mapping rate of each accession are listed in Additional file 2. Cultivated rice comprises two major subspecies, known as the *indica* and *japonica* subspecies. The *indica* subspecies contains *indica* and *aus* subgroups and the *japonica* subspecies contains temperate *japonica* and tropical *japonica* subgroups (Figure S1a in Additional file 3) [8]. We classified these 1483 accessions into four divergent groups, corresponding to the four subgroups of cultivated rice, based on principal component analysis using SNPs (“Materials and methods”). The mapping rates of the *indica* accessions were much lower than those of the *japonica* accessions (Figure S1b in Additional file 3), mainly because the Nipponbare reference accession is a *japonica* rice cultivar. This result indicated that a lot of dispensable sequences were present in some rice accessions but absent from the Nipponbare reference accession.

**Fig. 1:**
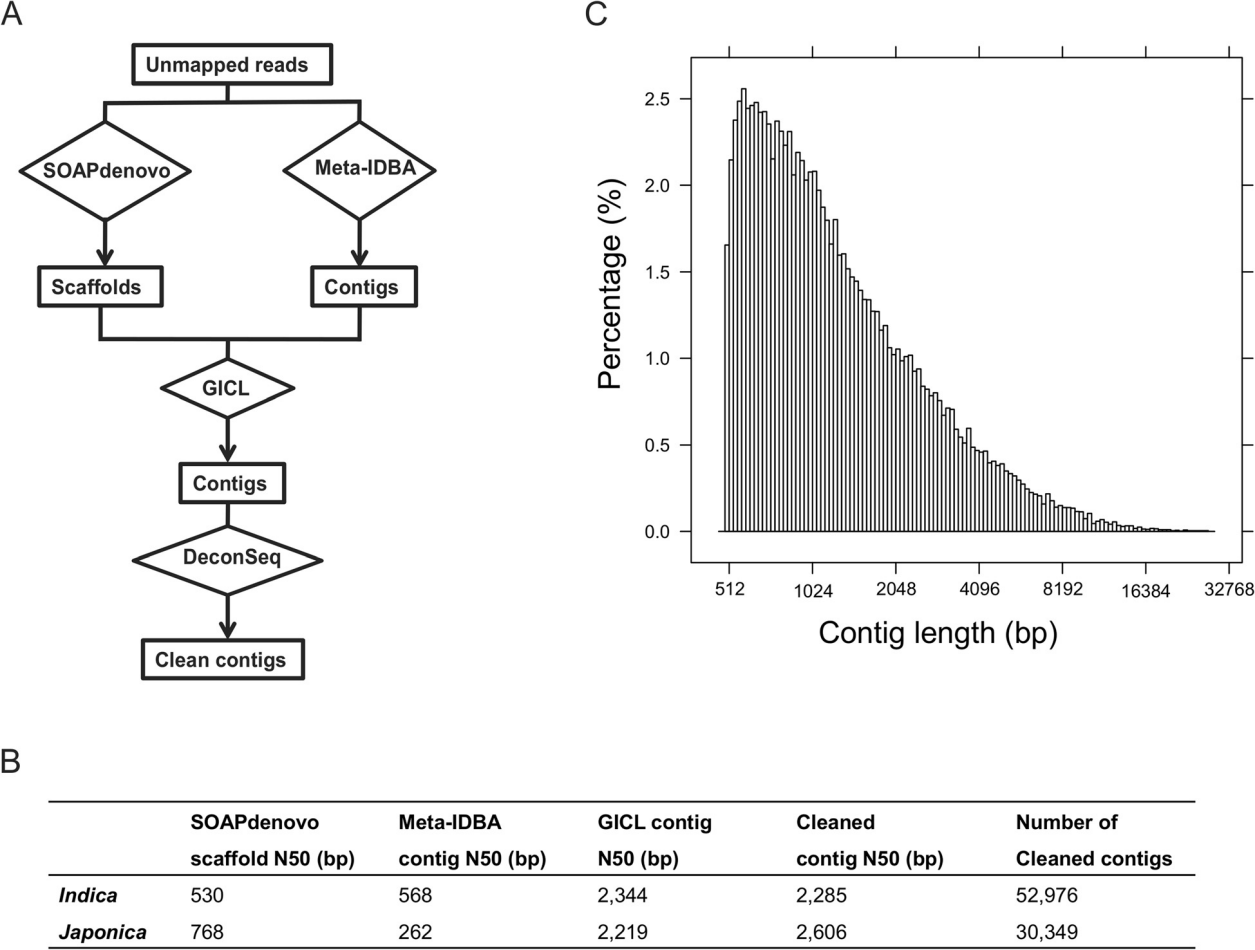
Assembly strategy and assembly results for the *indica* and *japonica* dispensable genomes. **a** The strategy for de novo assembly of low-coverage sequencing data of multiple accessions. *Unmapped reads* are read pairs of which at least one read could not be mapped to the Nipponbare reference genome. **b** Assembly results using different tools. **c** The length distribution of contigs of the *indica* dispensable genome

We proposed an assembly strategy incorporating SOAPdenovo [21], Meta-IDBA [22], and GICL [23] to assemble the dispensable sequences (“Materials and methods”; Fig. 1a). Both reads of a read pair of which at least one read could not be mapped to the reference genome were extracted to do de novo assembly (“Materials and methods”). In total, 395,891,829 read pairs (7.0 % of all the read pairs) were collected, of which 628,163,124 reads (79.3 %) could not be mapped to the Nipponbare genome (release 6.1 of the MSU Rice Genome Annotation Project). Due to the substantial genomic differences between the *indica* and *japonica* subspecies, independent assembly was carried out using reads of accessions belonging to the two subspecies respectively. The reads of all *indica* accessions were assembled using SOAPdenovo and Meta-IDBA independently. The scaffolds generated by SOAPdenovo and the contigs produced by Meta-IDBA for the *indica* subspecies were further clustered and then assembled using GICL. The reads of the *japonica* accessions were assembled using the same strategy. Contig N50 of the assembly of both subspecies was greatly improved using GICL compared with the assembly results of SOAPdenovo and Meta-IDBA (Fig. 1b). Contig N50 of the final assembly of the *indica* subspecies was 2344 bp, which was close to the average length of non-transposon loci of the rice Nipponbare reference genome (2853 bp) [17]. The final assembly of the *indica* subspecies, after the removal of contaminations (“Materials and methods”), contained 52,976 sequences with an average length of 1677 bp (ranging from 500 bp to 26,940 bp) while the assembly of the *japonica* group was composed of 30,349 sequences with an average length of 1877 bp (ranging from 500–28,300 bp) (Fig. 1c). These two assemblies represent the *indica* dispensable genome and the *japonica* dispensable genome, respectively. Only a total of 6397 sequences (12.1 %) of the *indica* dispensable genome and 3315 sequences (10.9 %) of the *japonica* dispensable genome were aligned to the Nipponbare genome (release 6.1 of the MSU Rice Genome Annotation Project) with ≥85 % coverage and ≥85 % identity.

**Fig. 2:**
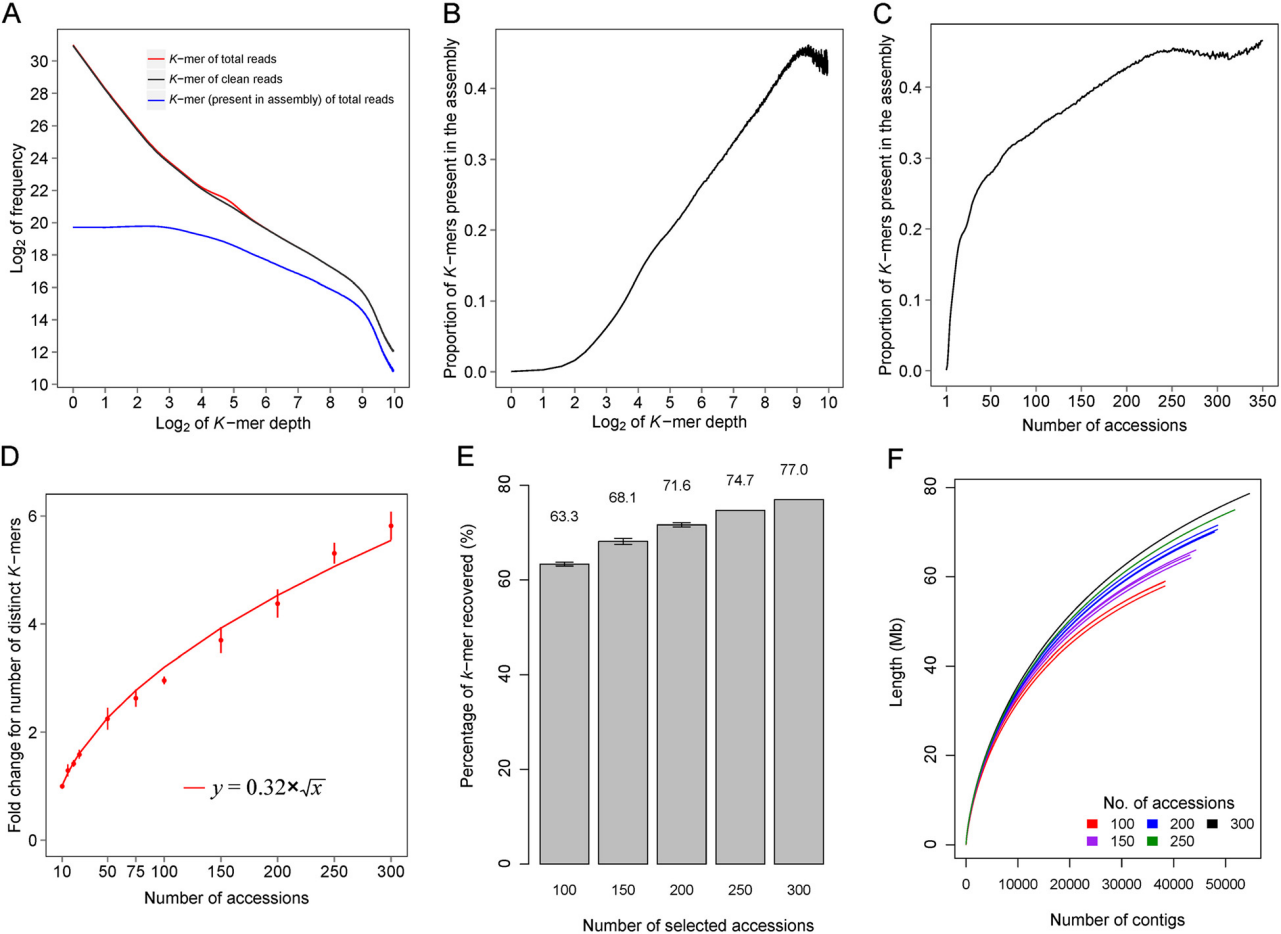
Evaluation of the assembly strategy by investigating *k*-mer distributions of reads of the *indica* accessions and the assembly result with randomly sampled accessions. **a** Number of *k*-mers with different depth in total sequencing reads and clean reads. **b** Proportion of *k*-mers present in the final assembly for *k*-mers with differing depth in all the sequencing reads. **c** Proportion of *k*-mers present in the final assembly for *k*-mers that are found in various numbers of accessions. **d** Number of *k*-mers with specific depth in reads of randomly sampled accessions. Each sampling was conducted with five replicates. The y-axis, *Fold change for number of distinct* k*-mers*, is the number of distinct *k*-mers in different numbers of sampled accessions divided by that of ten randomly sampled accessions. *Vertical bars* represent the standard deviation. The *red line* is the fitting curve determined by regression modeling. **e** The proportion of *k*-mers in the *indica* final assembly recovered by the assembly of randomly sampled rice accessions. The assemblies of 100, 150, and 200 accessions were generated with three replicates while the assemblies of 250 and 300 accessions were generated without replicates. Error bars represent the standard deviation. **f** The number of contigs and the length of all the contigs for each assembly result. All the assemblies were generated using the approach described in the “Materials and methods”, without the removal of contaminations by aligning to the NCBI Nucleotide collection database

We evaluated this assembly strategy by investigating *k*-mer distributions in reads used to build the dispensable genome and in the final assembly. A *k*-mer length of 19 was chosen to guarantee that the majority of *k*-mers were unique while keeping computing resource requirements relatively low. About 90.9 %, 85.2 %, and 86.4 % of all 19-mers of the Nipponbare reference genome and the *indica* and *japonica* dispensable genomes, respectively, were unique according to results from Jellyfish [24]. A total of 2.97 billion 19-mers were detected in reads used to build the *indica* dispensable genome, 71.9 % of which were observed only once (Fig. 2a). We did not observe any peak in the distribution of 19-mers, which was quite distinct from that of deep sequencing reads of a single accession. The frequency of 19-mers decreased, along with an increase in depth, in both the sequencing reads and the cleaned reads (Fig. 2a). The depth of 91.0 % of all 19-mers was smaller than half of the average *k*-mer depth of all 19-mers (7.05×). This is probably due to the high heterozygosity in the reads used to assemble the dispensable genome as we mixed reads of hundreds of accessions to perform the assembly. In all, 66,135,176 19-mers were detected in the *indica* assembly (Fig. 2a). The proportion of 19-mers present in the final assembly increased along with the increasing 19-mer depth of all the sequencing reads (Fig. 2b); however, the rate of increase slowed as the depth increased. Only 0.04 % of the 19-mers observed once in the sequencing reads were present in the *indica* assembly while more than 30.0 % of the 19-mers with a depth greater than 100 were in the assembly (Fig. 2b). To reduce the influence of different sequencing depth among accessions, we did additional analysis based on 353 *indica* accessions out of the 533 rice accessions with similar sequencing depth of ~2.5× (Additional file 1). We observed that 19-mers present in the sequencing reads of more accessions were more frequently found in the final assembly (Fig. 2c), as about 0.1 % of the 19-mers present in only one accession and 34.1 % of the 19-mers present in 100 accessions were found in the final assembly, respectively.

We further sampled randomly from the 353 *indica* accessions and investigated the distribution of 19-mers in the sequencing reads of the sampled accessions. To minimize the interference of sequencing error, we only analyzed *k*-mers with depth ≥2 in the sampled reads, the number of which continuously increased along with the increase in the number of accessions (Fig. 2d). However, the increment rate of the distinct *k*-mer number was lower than that of the number of sampled accessions, as the number of distinct *k*-mers with depth ≥2 was shown to scale with the square root of the number of sampled accessions (as revealed by regression modeling; *R*^2^ = 0.998, *P* = 1.78 × 10^−14^, Fig. 2d). We also generated assemblies using sequencing reads of randomly selected accessions using the same assembly strategy to build the dispensable genomes. The number of 19-mers of the *indica* dispensable genome was recovered cumulatively by the assembly with an increasing number of accessions (Fig. 2e). The number of contigs and the total length of the assembly also increased with increasing number of accessions used (Fig. 2f). The 19-mer distribution of the sequencing reads of the *japonica* accessions was similar to that of the *indica* accessions with only little difference (Figure S2a–c in Additional file 3). About 0.09 % of 19-mers observed only once while more than 41.9 % of 19-mers observed ≥100 times in the sequencing reads were present in the *japonica* assembly (Figure S2b in Additional file 3). About 83.5 % of all the 19-mers were observed with depth smaller than half of the average depth of all 19-mers (2.85×), a proportion smaller than that of all the *indica* reads. Since the genome diversity of *indica* accessions is higher than that of *japonica* accessions [25], these observations indicate that low sequence diversity was beneficial to the assembly of population sequencing data. Taken together, these results demonstrate the feasibility of constructing the rice dispensable genome based on assembly of population sequencing data and provide helpful information for experimental design of future studies on population sequencing data assembly.

### Evaluation of the assembly results

**Fig. 3:**
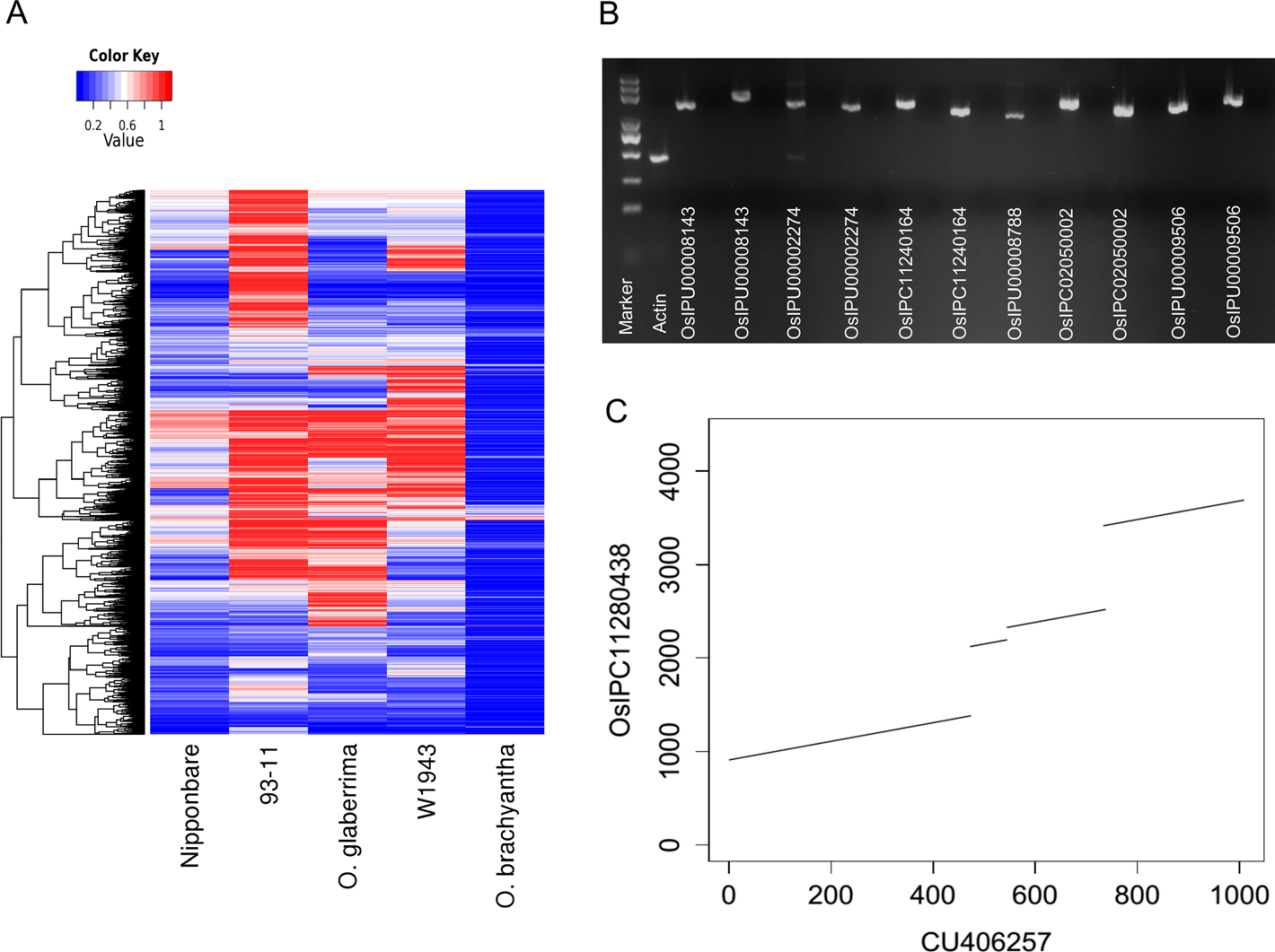
Evaluation of the assembly result. **a** Heatmap of the query coverage of alignment of each contig of the *indica* dispensable genome to different *Oryza* genomes. Each row represents a contig and each column denotes a sequenced genome. Only the contigs that could be aligned to the non-reference genome with improved match length (≥100 bp longer) compared with the alignment to the Nipponbare reference genome are shown. *O. brachyantha* and *O. glaberrima* are both *Oryza* species, *Nipponbare* is the rice reference genome, *93-11* is a typical rice variety belonging to the *indica* subspecies, and *W1943* is a representative accession of *Oryza rufipogon*. **b** PCR validation of 11 DNA fragments from the randomly selected contigs of the *indica* dispensable genome. For each DNA fragment, two PCR results (two adjacent lanes) are shown. The lanes marked with contig names are PCR results using DNA of the selected rice accessions while the unmarked lanes are PCR results using DNA of Nipponbare (see Additional file 5 for more details). Lanes marked with “*Actin*” are the PCR results with actin using DNA of the Nipponbare accession. The amplification sizes of markers are indicated in Fig. S4 in Additional file 3. **c** A dot plot showing the Blastn alignment result of an O. *rufipogon* gene (CU406257) to a contig (OsIPC11280438) of the *indica* dispensable genome

To evaluate the quality of the assembly results, all the contigs were aligned to five sequenced genomes of the *Oryza* genus — *Oryza sativa* L. ssp. *japonica* (Nipponbare), *Oryza sativa* L. ssp. *indica* (*93-11*) [26], *Oryza glaberrima* (African cultivated rice) [27], *Oryza brachyantha* (African wild rice) [28], and *Oryza rufipogon* (W1943, Asian wild rice) [29] — using Blastn (with parameter “-F F”). A total of 30,440 (57.5 %) contigs of the *indica* dispensable genome were aligned to the other four sequenced *Oryza* genomes with improved match length (≥100 bp longer) compared with the alignment to the Nipponbare genome (Fig. 3a; Additional file 4). Although the remaining 22,536 contigs (42.5 %) were not aligned to the other four *Oryza* genomes with remarkable improved match length, 17,956 (79.7 %) of them were aligned with higher alignment scores and 11,378 (63.4 %) of them were aligned with improved identity (≥1 % higher) to the other sequenced *Oryza* genomes compared with the alignment to the Nipponbare genome (Figure S3 in Additional file 3). Using a more stringent rule, 8932 (16.8 %) contigs were aligned to the other four sequenced *Oryza* genomes with improved match length (≥90 % of the length of the contig) compared with the alignment to the Nipponbare genome (≤50 % of the length of the contig).

To further evaluate the assembly results, 43 pairs of PCR primers were designed to amplify DNA fragments from the 30 randomly selected contigs (Additional file 5). All of the 43 fragments were amplified with expected sizes using genomic DNA of the corresponding rice accessions which were predicted to contain the sequences, while none could be amplified using genomic DNA of Nipponbare (Fig. 3b; Figure S4 in Additional file 3).

To examine whether the assembly can successfully construct the sequences of genes reported to be absent from the Nipponbare genome, we collected ten such genes from the published literature (Table 1), all of which were related to important traits, including grain size (*GW5* [13, 30]), disease resistance (*Pikm1-TS* [15], *Pikm2-TS* [15], *Pib* [31], and *Xa27* [32]), water logging resistance (*SNORKEL1*, *SNORKEL2* [33], and *Sub1A* [14]), salt tolerance (*OsHKT2* [34]), and phosphorus-deficiency tolerance (*PSTOL1* [35]). The sequences of these genes were aligned to the dispensable genomes using Blastn and most of these genes were successfully constructed in the dispensable genomes (Table 1), except *SNORKEL1* and *SNORKEL2*, which are found uniquely in deepwater rice not included in the accessions we used to build the dispensable genomes, and *Pib* (longer than 10 kb), only a portion of which was assembled.

**Table 1:** Alignment results of the cloned genes absent from the Nipponbare genome to the dispensable genomes

Gene name	Length (bp)	Trait	Reference	Raw assembly		Local de novo reassembly		
				Query	Identity (%)	Query	Identity (%)	Number of haplotypes
				coverage (%)		coverage (%)		
*GW5*	435	Grain width	Shomura et al. 2008 [13]	100	90	100	100	5
			Weng et al. 2008 [30]					
*Sub1A*	1663	Submergence tolerance	Xu et al. 2006 [14]	100	99	100	100	7
*Xa27*	342	Blight resistance	Gu et al. 2005 [32]	100	100	100	100	16
*PSTOL1*	975	Phosphorus starvation tolerance	Gamuyao et al. 2012 [35]	100	97	100	100	13
*Pikm1-TS*	6319	Blast resistance	Ashikawa et al. 2008 [15]	100	99	17	99	3
*Pikm2-TS*	3229	Blast resistance	Ashikawa et al. 2008 [15]	100	99	100	99	7
*OsHKT2*	1781	Na^+^- and K^+^-coupled transporter	Horie et al. 2001 [34]	95	95	62	97	19
*Pib*	10,322	Blast resistance	Wang et al. 1999 [31]	33	80	-	-	-
*SNORKEL1*	1487	Deepwater response	Hattori et al. 2009 [33]	7	83	-	-	-
*SNORKEL2*	1179	Deepwater response	Hattori et al. 2009 [33]	12	82	-	-	-

*Length* is the length of the reported sequence of the gene. *Query coverage (%)* is the percentage of the reported sequence matched by a dispensable sequence from raw assembly or local de novo reassembly. *Identity (%)* is the percentage of the reported sequence identical with a dispensable sequence from raw assembly or local de novo reassembly. *Number of haplotypes* is the number of haplotypes constructed with the local de novo reassembly

*PSTOL1* was identified in the traditional *aus*-type rice variety Kasalath [35]. The genomic region of Kasalath harboring *PSTOL1* differs greatly from that of Nipponbare. The sequences of Kasalath in this region were decoded in a previous study [36]. We found that many (48.7 %) genes predicted in this region were present in the assembled result of the *indica* or the *japonica* dispensable genome (Additional file 6).

Previous studies reported that full-length cDNA sequences of 15 O. *rufipogon*-specific genes could not be mapped to either the Nipponbare or *93-11* genome [37, 38], and that five of these genes were up- or down-regulated in wounding and/or submergence conditions. We found that 9 of these 15 genes were present in the *indica* or *japonica* dispensable genome (Blastn, query coverage ≥80 %, identity ≥90 %; Additional file 7; Figure 3c).

The redundancy between the *indica* and the *japonica* dispensable genomes was evaluated by aligning the *japonica* dispensable genome to the *indica* dispensable genome using Blastn: 18,033 contigs (34 %) of the *indica* dispensable genome had alignment hits to the *japonica* dispensable genome (identity ≥90 %, match length ≥60 % of the length of the *indica* contig) and 16,426 contigs (54 %) of the *japonica* dispensable genome had alignment hits to the *indica* dispensable genome (identity ≥90 %, match length ≥60 % of the length of the *japonica* contig). In total, 7820 contigs of the *indica* dispensable genome corresponding to 7689 contigs of the *japonica* dispensable genome (a total of 8209 pairs) were found to have reciprocal coordinate overlap of more than 60 % (identity ≥90 %). These results suggest that most of the sequences of the dispensable genome are subspecies-specific and subject to rapid gain and loss.

### Annotation of protein-coding genes and transposable elements in the dispensable genomes

We utilized an annotation pipeline incorporating Fgenesh [39], AUGUSTUS [40], GeneWise [41], PASA [42] and EvidenceModeler [43] to predict protein-coding genes in the dispensable genomes. For the *indica* dispensable genome, 8991 protein-coding genes were predicted, 1913 of which with strong evidence of expression or homology were tagged as high confidence genes (“Materials and methods”). For the *japonica* dispensable genome, 6366 protein-coding genes were predicted, of which 1120 were tagged as high confidence genes.

About 186 predicted proteins of the *indica* dispensable genome were homologous to cloned genes, including disease- or stress-resistance genes (Blastp, E-value < 1e-10; Additional file 8) [12, 44], such as the *Brown planthopper* resistance gene *Bph14*, the blast resistance gene *Pid3* and the starch branching enzyme IIb gene *BEIIb* [45] (Figure S5 in Additional file 3). These genes may provide important candidates for future crop improvements.

Among seven genes reported missing from the reference genome, the structures of the two rice blast resistance genes *Pikm1-TS* and *Pikm2-TS* were accurately annotated. Genes *Sub1A* and *OsHKT2* were correctly identified but the predicted structures were inconsistent with those in previous reports [14, 34].

**Fig. 4:**
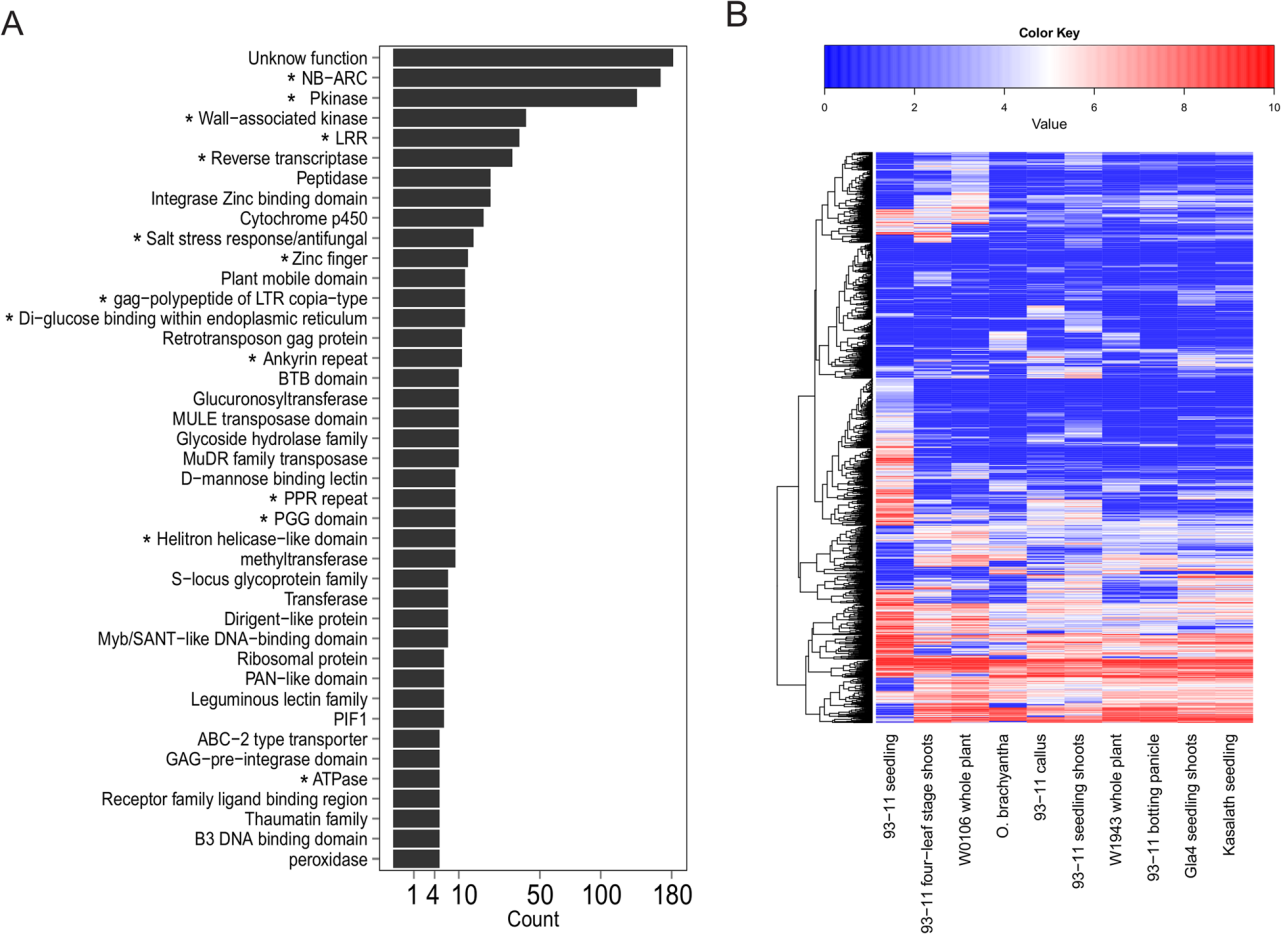
Function annotation of predicted proteins of the *indica* dispensable genome and the expression profile of predicted genes. **a** Annotation of protein domains and families was done using Pfam. Domains and families enriched in the dispensable genome are indicated by an *asterisk*. The x-axis is logarithmic scaled. **b** Expression values (log2 of RPKM) of 2257 predicted genes in ten RNA-seq experiments

Protein domains and families coded by these 1913 high confidence genes were annotated using Pfam [46]. More than 45 % of these genes are members of gene families (Additional file 9). Besides transposon-related domains, leucine-rich repeat (LRR), NB-ARC domain, zinc finger transcription factors, the stress-antifung family, and protein kinase family proteins were found significantly enriched in the dispensable genome compared with those in the reference genome (Fisher’s exact test, *P* ≤ 1 × 10^−4^; Fig. 4a). Of the 1913 predicted genes, 1489 were found to be expressed [reads/per kilobase merged exonic region/per million mapped reads (RPKM) ≥4] using RNA-seq data of ten rice varieties/tissues (Fig. 4b; Additional file 10).

Transposons were reported to play important roles in genome evolution [47]. For the *indica* dispensable genome, 23,644 transposons were found in 15,542 contigs (29.3 % of all the contigs; Additional file 11). These transposons were categorized into three major classes, retrotransposons, DNA transposons and miniature inverted-repeat transposable elements (MITEs), and different subclasses based on the classification methods used by the Plant Repeat Databases at MSU [48] (Figure S6 and S7 in Additional file 3). The transposon compositions of the *indica* and the *japonica* dispensable genomes exhibited only slight differences (Figure S6 and S7 in Additional file 3; Additional file 11). Most of the annotated transposons were DNA transposons and MITEs. The majority of DNA transposons were *harbinger-*like elements, a member of which was reported to have retained its biological activity in the coelacanth genome [49]. Most of the MITEs belong to the *Stowaway* group, which were found to be active in some plant genomes [50, 51].

### Determining genomic positions of dispensable sequences relative to the Nipponbare reference genome

In studies of QTL mapping and association mapping, causal genes were usually mapped to a narrow genomic region in which candidates were picked up by the researchers based on gene annotation of the reference genome. In cases where the causal gene does not exist in the reference genome, however, such attempts would be futile. Thus, determining genomic positions of dispensable sequences relative to the Nipponbare reference genome would be helpful for picking candidates for future QTL mapping and association mapping studies in rice, as these provide additional possible candidates.

**Fig. 5:**
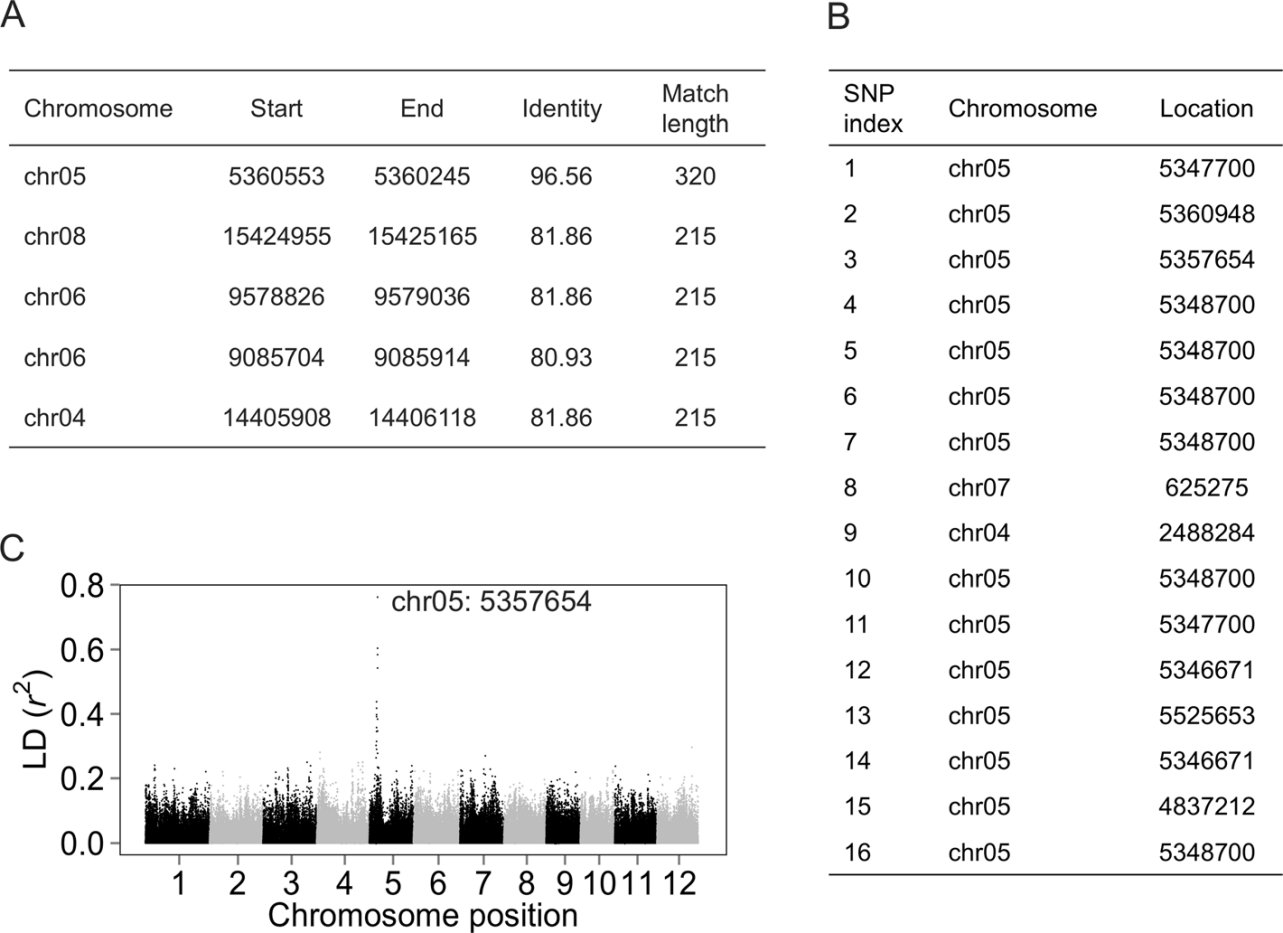
The approach, based on the integration of alignment results and linkage disequilibrium (LD), to assign each dispensable sequence a genomic position relative to the Nipponbare reference genome. **a** The top five Blast hits for the contig harboring *GW5* when aligned to the Nipponbare genome. **b** Chromosome locations most tightly linked with the contig harboring *GW5* determined by the 16 SNPs of this contig. **c** The LD between a specific SNP (the third SNP in (**b**)) of the contig harboring *GW5* and the 280,000 SNPs selected from whole genome SNPs. The SNP with the highest LD has chromosome location chr05: 5357654

Since the reads used to construct contigs of the dispensable genome contained hanging read pairs (of which only one read could be reliably aligned to the Nipponbare reference genome), many contigs contained a portion of sequences which could be found in the reference genome. To obtain the genomic position of the contig relative to the Nipponbare genome, each contig was aligned to the Nipponbare reference genome and hits with the top three highest scores were retained (Fig. 5a). However, this was not suitable for contigs with repetitive sequences.

As a result, a linkage disequilibrium (LD)-based approach was applied to identify chromosome positions of the reference genome that were most tightly linked with each contig. The determined chromosome position was considered as the approximate genomic position of this contig relative to the Nipponbare genome. All the 11.3 billion paired-end reads of the 1483 rice accessions were mapped to the dispensable genome using BWA (version 0.6.1) and then SNPs were called using SAMtools (version 0.1.17) [20, 52]. Genotypes of all the rice accessions were obtained at 280,000 evenly distributed SNP sites from RiceVarMap [18, 53], a genomic variation database constructed based on the same set of sequencing data used in this study. The LD (*r*^*2*^) between a specific SNP on a contig and all the 280,000 SNPs were calculated and the chromosome location of the SNP in the Nipponbare genome with the highest LD was regarded as the chromosome location of this contig determined by this specific SNP (Fig. 5b, c). Each SNP in a contig determined a chromosome location most tightly linked with this contig. The frequency of each chromosome location of a contig was calculated by measuring each location in a 100-kb unit. The two chromosome locations with the highest frequency and the second highest frequency were recorded.

The results from the alignment and LD-based approaches were integrated to obtain the genomic position of each contig relative to the Nipponbare reference genome (Additional file 12). For contigs with results from just a single approach, the best placement was taken as the final location only if it was overwhelmingly superior to the second best placement (Additional file 12). For contigs with locations reported by both approaches within 1 Mb of each other, the result based on the alignment was considered as the final location (Additional file 12). For contigs for which the two approaches gave conflicting results, we adopted the results of the LD-based approach only if it met the set criteria (Additional file 12). For the *indica* dispensable genome, 33,056 (62.4 %) contigs were assigned genomic positions with consistent results between the two approaches, the two approaches gave conflicting results for 3749 (7.1 %), 3975 (7.5 %) contigs were assigned genomic positions by the LD-based approach alone, and 657 (1.2 %) contigs were assigned genomic positions based solely on the alignment results. The positions of the remaining 11,539 (21.8 %) contigs failed to be assigned.

All of the genes that were reported to be absent from the reference genome and constructed in the dispensable genome were assigned correct genomic positions (“Materials and methods”) relative to the Nipponbare reference genome except for *Sub1A* (Table 2). We further dissected the reason why *Sub1A* could not be assigned a genomic position. Few SNPs between different accessions were identified in the contig harboring *Sub1A*. The frequency for the most tightly linked chromosome location was not overwhelming when compared with the frequency for the second tightly linked chromosome location (2 to 1). What is more, *Sub1A* contains a short repetitive sequence (930 bp) that can be aligned to multiple chromosome locations. Nevertheless, the most tightly linked chromosome location determined by the LD-based approach was only 130 kb away from the actual location of *Sub1A* relative to the reported location in the Nipponbare genome. This demonstrates the efficiency of this approach to determine the genomic positions of sequence insertions in the reference genome. The two most tightly linked chromosome locations and the two best alignment results for each contig in the *indica* dispensable genome are shown in Additional file 13.

**Table 2:** Locations of the contigs which harbor cloned genes that are absent from the Nipponbare genome

Gene	Genomic position from literature	Assigned genomic position	Minimum distance (bp)^a^	Contig
*GW5*	Chr05:5360552-5360941	Chr05:5360245-5360553	0	OsIPC05060021
*Sub1A*	Chr09:6403884-6404699	Not assigned	Not assigned	OsIPC09070023
*Xa27*	Chr06:23653458-23654407	Chr06:23634625-23634930	18,528	OsIPC06240124
*PSTOL1*	Chr12:15874815-15875229	Chr12:15874535-15874748	67	OsJPC12160175
*OsHKT2*	Not assigned	Chr06:29552379-29552608	Not assigned	OsJPC06300057
*Pikm1-TS*	Chr11: 27506909-27507692	Chr11:27511712-27511965	4020	OsIPC11280248
*Pikm2-TS*	Chr11:27515464-27517260	Chr11:27519921-27520230	2661	OsIPC11280253

^a^ The minimum distance between the genomic position relative to the Nipponbare genome identified from the literature and the assigned genomic position

**Fig. 6:**
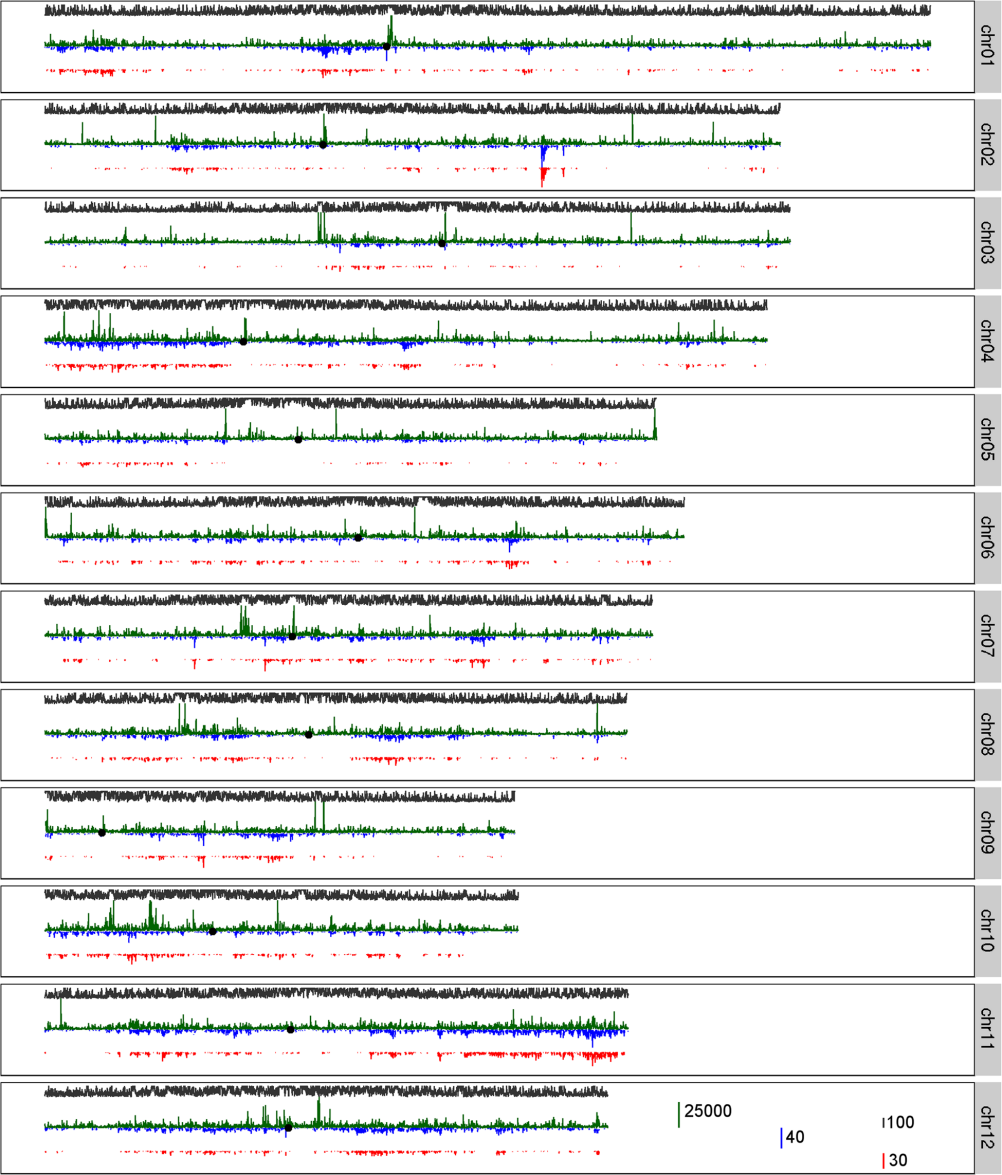
The chromosome distribution of contigs of the dispensable genome and hanging reads. The 12 chromosomes are arranged from top to bottom. The height of the *dark green bars* denote the number of read pairs of which only one read can be mapped to the reference genome (hanging reads) in each chromosome window (10 kb). The height of the *blue bars* denotes the number of assembled contigs of the *indica* dispensable genome while the height of the *red bars* denotes the number of assembled contigs of the *japonica* dispensable genome in each chromosome window (10 kb). The height of the *grey bars* denotes the sequence repeat density represented as the percentage of sequences masked due to repetitiveness in each chromosome window (10 kb). The *black points* represent the chromosome locations of 12 centromeres

The chromosome distributions of contigs of the *indica* and *japonica* dispensable genomes were quite similar (Fig. 6). The sequence of each contig of the *indica* dispensable genome was aligned to all the contigs of the *japonica* dispensable genome that were located within 50 kb of the *indica* contig. The sequences of most of these *indica*–*japonica* contig pairs were quite distinct (Figure S8 in Additional file 3), implying that the similarity between the chromosome distributions of the *indica* and *japonica* dispensable genomes was not caused by the sequence similarity between the *indica* and *japonica* contigs located in nearby regions. Of the 8209 *indica*–*japonica* contig pairs which were found to have reciprocal coordinate overlap of more than 60 % (identity ≥90 %), both contigs of 6316 contig pairs were assigned genomic positions and the positions of the two contigs for 5279 contig pairs were within 100 kb of each other (Additional file 14).

We further inspected the chromosome distribution of potential insertions to the Nipponbare genome by examining the locations of all the hanging read pairs. The chromosome distribution of contigs was in accordance with the distribution of these potential insertions in general (Fig. 6). Hanging read pairs were found to be enriched around the centromeres of nine chromosomes and the telomeres of two chromosomes (Fig. 6). This may be caused by sequence gaps in the regions of the reference genome. Several chromosome locations that were neither centromeres nor telomeres were also found enriched with hanging read pairs. Although large numbers of hanging read pairs were found in dozens of genomic regions with high repeat density, hotspots of hanging read pairs were not necessary in genomic regions with high repeat density (Fig. 6). As a result, 833 chromosome insertion hotspots were identified based on the chromosome distribution of hanging read pairs and that of contigs of the dispensable genome (Additional files 12 and 15).

### Local de novo reassembly for each contig of the dispensable genome

We observed that the raw contigs of the assembly were not quite accurate as a result of sequence heterozygosity introduced by mixing of the reads of multiple accessions. Although the sequence of *Sub1A* was successfully constructed, five SNPs were detected between the assembled sequence and the reported sequences of different haplotypes of *Sub1A*. In order to obtain an accurate assembly result for each haplotype, a haplotype-based de novo assembly strategy proposed by Huang et al. [8] was adopted with slight modification to perform local assembly for each contig (“Materials and methods”).

To evaluate the effects of this local assembly strategy, 1000 genomic regions with an average length of 5322 bp (ranging from 503 bp to 9994 bp) were randomly selected and the read pairs of all the 1483 accessions mapped to these regions were extracted and grouped by local SNPs to perform local reassemblies. For each region, the longest assembly was chosen as the best assembly. The match length between the best assembly result and the original region was used to evaluate the assembly performance. For more than 58 % of these regions, the match length between the best reassembly and the original region was larger than 70 % of the length of the original region. Most of the regions (243 regions, 24.3 %) with poor reassembly results were found to be in the repeat regions of the genome (Figure S9a, c in Additional file 3), which had multiple Blastn alignment to the reference genome (query coverage ≥30 %, identity ≥80 %), while most regions (756, 75.6 %) with good reassembly results were located in unique genomic regions (Figure S9e, g in Additional file 3). A few regions (27, 2.7 %) located in unique genomic regions were poorly assembled because they were relatively long and the reads used to build the best assembly were obtained from relatively more accessions (Figure S9i–l in Additional file 3). In contrast, some regions (61 regions, 6.1 %) in the repeat part of the genome were well reassembled because they were relatively short and the reads used to build the best assembly were obtained from relatively fewer accessions (Figure S9m–p in Additional file 3).

Using this strategy, the sequences of at least one haplotype of *GW5*, *Sub1A*, *Xa27*, and *PSTOL1* reported in previous studies [14, 30, 32] were accurately built (Table 1; Figure S10 in Additional file 3). The local de novo assembly results for *Pikm1-TS*, *Pikm2-TS*, and *OsHKT2* were slightly different from the reported sequence, which may correspond to unreported haplotypes.

For the *indica* dispensable genome, 41,376 contigs which were assigned genomic positions relative to the Nipponbare genome were reassembled using local SNPs. About 70 % of them were reassembled with four to seven haplotypes (Figure S11 in Additional file 3). For about half of these 41,376 contigs, the longest local assembly was longer than the original contig. (Figure S11 in Additional file 3). The longest local assembly of about 5492 contigs of the *indica* dispensable genome were longer than the average length of the non-transposon loci of the rice Nipponbare reference genome (2853 bp) [17].

### Population pattern of sequences of the dispensable genome

To explore the population patterns of the dispensable sequences, all the reads of the 1483 accessions that were not aligned to the Nipponbare reference genome were mapped to the dispensable genomes. The population composition of each contig was determined as the number of reads from accessions belonging to each of the three major subpopulations (*indica*, *aus*, and *japonica*) classified by whole genome SNPs, divided by the total number of the reads mapped to this contig (Figure S12 in Additional file 3). Great disparities were found in the population composition of reads aligned to different dispensable sequences. For 29,466 sequences of the *indica* dispensable genome, more than 80 % of the aligned reads were from a single subgroup. Similarly, 11,914 sequences of the *japonica* dispensable genome were composed of reads (≥80 % of total reads) mainly from a single subgroup. As the number of rice accessions and the sequencing coverage for each subpopulation were not uniform, the population composition of each contig was normalized through dividing the number of reads of each subpopulation by the total sequencing coverage of the corresponding subpopulation (Figure S12 in Additional file 3). A sequence was determined as subpopulation-preferred if the normalized proportion of mapped reads belonging to a specific subpopulation was larger than 0.8. We found 4879 of the contigs of the *indica* dispensable genome to be *indica*-preferred and 4211 were *aus*-preferred (Additional file 16) while only 80 were *japonica*-preferred. For 32,748 (61.8 %) *indica* dispensable sequences, the normalized proportion of mapped reads from *japonica* accessions was less than 0.1. Of all the *japonica* dispensable sequences, 1731 were *japonica*-preferred, 1453 were *indica*-preferred and 771 were *aus*-preferred. For only 4709 (15.5 %) *japonica* dispensable sequences was the normalized proportion of mapped reads from *indica* accessions less than 0.1. These results suggest that lots of sequences might have introgressed from the *indica* subspecies to the *japonica* subspecies.

We further investigated the read coverage rate of dispensable sequences for each subpopulation. For each contig of the dispensable genome, the read coverage rate was calculated by mapping raw reads of each accession to this contig and the highest read coverage rate for each subpopulation was taken as the read coverage rate for each subpopulation. The read coverage rates of all the three subpopulations for more than 40 % of the 52,976 contigs of the *indica* dispensable genome were higher than 80 % (Figure S13 in Additional file 3). The coverage rates of a lot of dispensable sequences were quite different among subpopulations. More sequences with higher coverage rates were found in *indica* than in *japonica* (Figure S13 in Additional file 3). This is consistent with the results of previous studies that the genome diversity of *indica* accessions is higher than the genome diversity of *japonica* [25].

The population pattern of each haplotype of the contigs in the dispensable genome was also examined. Interestingly, the sequence of *PSTOL1*, which was identified in the *aus*-type rice variety Kasalath, could be constructed accurately using reads from *japonica* accessions [35]. This indicates the complexity of the population patterns of the dispensable genome.

### The composition of dispensable sequences

To investigate the composition of dispensable sequences, we tried to explore the relationship between the dispensable sequences and the Nipponbare reference genome, especially the sequences of annotated genes. The sequences of exons and introns with a length greater than 200 bp were aligned to the contigs of the dispensable genomes using Blastn. If multiple exons/introns that were aligned to a contig overlapped with each other, only the one with the largest query coverage was retained. Only those exons/introns with more than 90 % of their sequences aligned to a contig were used for further analysis. In total, 5146 contigs of the *indica* dispensable genome contained multiple exons and introns from 6302 different reference genes (Figure S14a in Additional file 3; Additional file 17). Of all the 5146 contigs, 1165 (22.7 %) were aligned to the other sequenced *Oryza* genomes with improved match length (≥90 % of the length of the contig) compared with the alignment to the Nipponbare reference genome (≤50 % of the length of the contig), a proportion higher than the whole *indica* dispensable genome level (Fisher’s exact test, *P* ≤ 2.7e-24). Among these 6302 reference genes, 3893 were transposons or retrotransposons, 311 were annotated as expressed proteins, and 1101 were annotated as “hypothetical proteins” (release 6.1 of the MSU Rice Genome Annotation Project). The remaining 997 reference genes encoded proteins with potential functions, including OsWAK receptor-like protein kinase, disease resistance protein and jacalin-like lectin domain containing protein. Among these 6302 genes, genes encoding RNase, reverse transcriptase, protease, transposase, zinc finger, and plant mobile domain were found significantly enriched compared with the genes of the Nipponbare reference genome (Fisher’s exact test, *P* ≤ 1e-4; Additional file 18). For the *japonica* dispensable genome, 3120 contigs contained multiple exons and introns from 4278 different reference genes, among which genes encoding RNase, reverse transcriptase, protease, transposase, plant mobile domain, and salt stress response protein were found significantly enriched, which is quite similar to the *indica* dispensable genome.

The transposon composition of each contig of the *indica* dispensable genome was surveyed using the transposon annotation result provided by CENSOR [54]. If more than 50 % of the sequence of a contig was annotated to contain at least two transposons, it was considered as a transposon cluster. As a result, 636 transposon clusters were detected within the *indica* dispensable genome, 215 (33.8 %) of which were aligned to the other sequenced *Oryza* genomes with improved match length (≥90 % of the length of the contig) compared with the alignment to the Nipponbare genome (≤50 % of the length of the contig) (Figure S14b in Additional file 3). More than half of the transposons involved in the formation of these 636 transposon clusters were MITEs (Additional file 19). We also downloaded all the long terminal repeat (LTR) retrotransposons from RetrOryza and aligned the internal regions and LTR regions to the contigs of the dispensable genome [55]. An intact LTR retrotransposon contains two LTRs and an internal region. For the *indica* dispensable genome, 8668 (16 %) contigs were found to contain only one LTR or the internal region of a specific LTR retrotransposon and 896 (2 %) contigs contained at least two of the three elements of an LTR retrotransposon (Additional file 20). Among these 9564 contigs, 1198 (12.5 %) were aligned to the other sequenced *Oryza* genomes with improved match length (≥90 % of the length of the contig) compared with the alignment to the Nipponbare genome (≤50 % of the length of the contig).

In maize, rice, and *Arabidopsis*, a few *Mutator*-like transposable elements (MULEs) were shown to carry fragments of cellular genes, which were called Pack-MULEs. Jiang et al. [56] reported that over 3000 Pack-MULEs in rice contained fragments deriving from more than 1000 cellular genes. The *indica* dispensable genome was searched for Pack-MULEs following the procedures described by a previous study [57]. As a result, 35 Pack-MULEs were found to carry fragments of 37 cellular genes (Additional file 21). Twenty of these 37 cellular genes were annotated to encode hypothetical proteins while several genes were annotated to encode myb domain proteins and zinc finger proteins.

### Association mapping of agronomic and metabolic traits using the dispensable genome

Since the results of the population pattern analysis suggest that different rice accessions have different sequence compositions in their dispensable genomes, the differences in the dispensable sequences might be regarded as markers to investigate their contributions to phenotypic variation. Accordingly, we aligned all the reads unmapped to the Nipponbare genome to both the *indica* and *japonica* dispensable genomes and calculated the sequencing depth of each accession at every genomic position for each dispensable sequence (“Materials and methods”). Then each dispensable sequence was split into 500-bp overlapping windows (with 250-bp step size) and the average read depth in each window was calculated for each rice accession. Since the depth value for a specified 500-bp window varied among different accessions, it is referred to as a depth polymorphism (DP). The continuous data of DPs [termed continuous DPs (CDPs)] were transformed into Boolean values [termed Boolean DPs (BDPs)] (“Materials and methods”). A total of 463,765 DPs were constructed and 412,320 (88.9 %) were assigned genomic positions relative to the Nipponbare reference genome. Among the 1483 accessions, 533 accessions were collected and sequenced by us in a previous study with available phenotypic values for agronomic and metabolic traits [9]. We further carried out GWASs using both CDPs and BDPs.

**Fig. 7:**
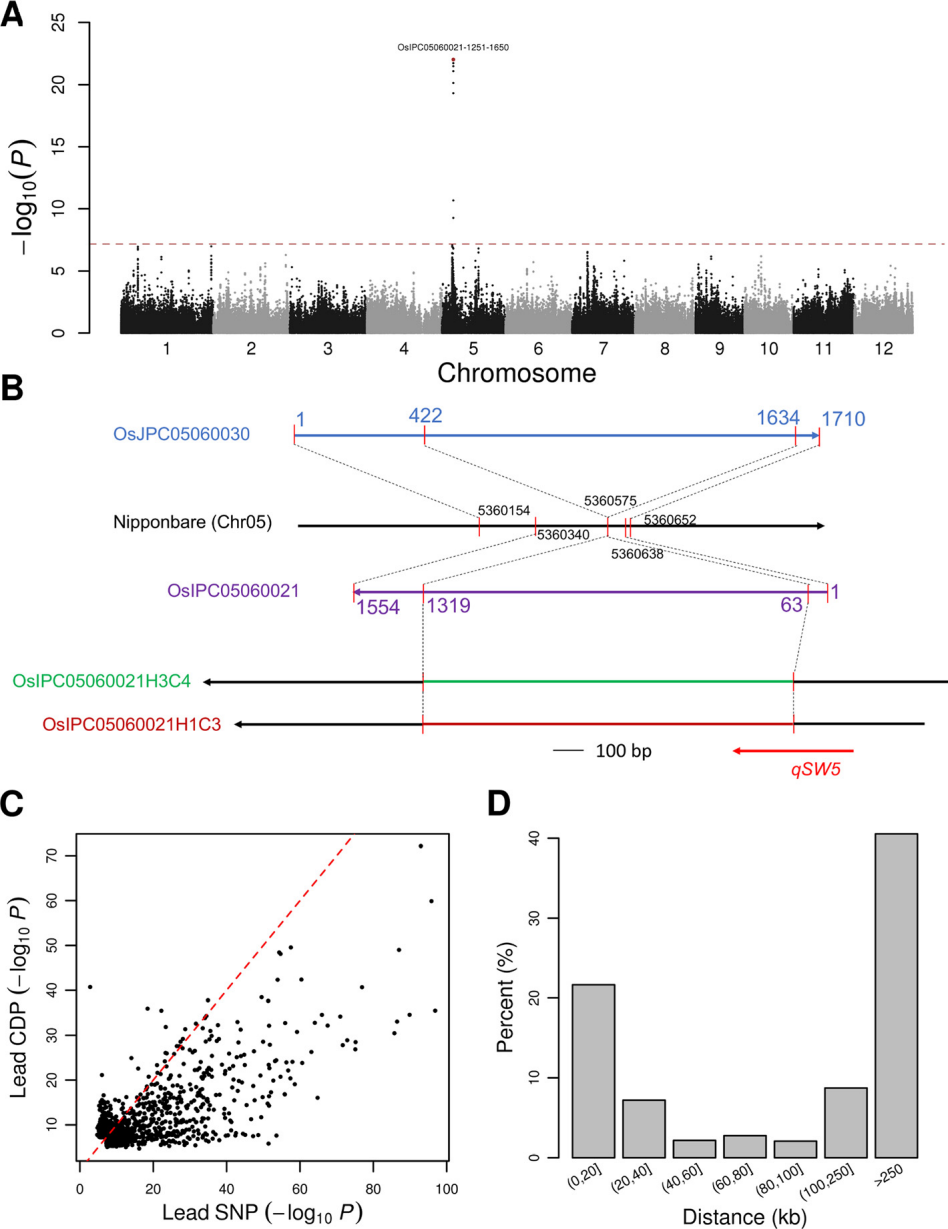
Comparisons of association mapping using linear mixed model based on reference and dispensable genomes. **a** Manhattan plots for association mapping of rice grain width using CDPs based on the dispensable genomes. The *horizontal dashed line* indicates the genome-wide significance threshold (*P* = 6.6 × 10^−8^). The lead CDP is marked in brown and labeled. **b** Alignment of both the raw assembly and the local reassembly of the contig harboring *GW5*/*qSW5* to the Nipponbare genome. The aligned regions of the contigs and the Nipponbare genome are indicated by *red bars* and connected by *dashed lines*. OsIPC05060021H3C4 and OsIPC05060021H1C3 are the local assembly of the contig OsIPC05060021. The *black parts* of these two contigs indicate the regions which could be aligned to the Nipponbare genome while the *green* and *red parts* denote insertion regions in the Nipponbare genome. The *arrows* indicate the strand of these sequences. The *red segment* of *qSW5* indicates the position of the coding region of *qSW5* in these sequences. **c** Comparisons of LMM *P* values of lead SNPs with that of lead CDPs for 1169 metabolic traits of which the *P* values for the lead SNPs or the lead CDPs passed the significance threshold. The *red dashed line* indicates where the *P* values of the two approaches are equal. **d** The distribution of chromosome distances between significant lead SNPs and the closest significant (*P* ≤ 6.6 × 10^−8^) or similarly significant (the difference of *P* values less than hundred times) CDPs

We first performed a GWAS for rice grain width using both a set of 3.9 million SNPs identified by mapping reads against the reference genome in a previous study [9] and the 0.46 million DPs (Fig. 7a, b). A sharp peak with a lead SNP, sf0505373357 (*P* = 1.3 × 10^−24^), 12 kb away from the cloned grain size gene *GW5* was identified (Figure S15a in Additional file 3). When using CDPs, the DP marker OsIPC05060021-1251-1650 (Fig. 7a) was most significant (*P* = 9.9 × 10^−23^) and another DP marker, OsJPC05060030-1-500, was the second most significant (*P* = 1.9 × 10^−22^). When using BDPs, these two markers became more significant (OsIPC05060021-1251-1650, *P* = 5.8 × 10^−37^; OsJPC05060030-1-500, *P* = 2.1 × 10^−36^). The first DP marker is from contig OsIPC05060021 of the *indica* dispensable genome and the second is from contig OsJPC05060030 of the *japonica* dispensable genome, both of which contain a large part of the *GW5*/*qSW5* coding region, which can be fully constructed by local de novo reassembly (Fig. 7b). *GW5* encodes a novel nuclear protein which is deleted from the Nipponbare genome and thus has not been annotated in any rice genome annotation (Fig. 7b).

We further carried out GWASs using linear mixed model (LMM) for rice metabolic traits, including 840 metabolites [9], using CDPs and BDPs. A set of 524 rice accessions were cultivated in two places and the collection of samples and metabolite profiling were performed separately; thus, the two replicates could be analyzed independently, generating a total of 1680 metabolic traits. After performing GWASs, the most significant SNP (termed the lead SNP), CDP and BDP for each trait were recorded and compared. In all, 854 traits with SNPs, 935 traits with CDPs and 790 traits with BDPs passed the genome-wide significance threshold (*P* = 6.6 × 10^−8^). Around 23.4 % (219/935) of the most significant CDPs and 11.4 % (90/790) of BDPs could not be located on the reference genome. Compared with the highest association signals identified using SNPs [9], we found that 23.5 % (220/935) and 16.6 % (131/790) of metabolites had stronger association signals (100 times smaller *P* values) with CDPs and BDPs, respectively (Fig. 7c; Figure S15c in Additional file 3). For 41.6 % of the 854 traits associated with significant SNPs, the most significant SNPs had concordant genomic locations (<100 kb) with significant CDPs (*P* ≤ 6.6 × 10^−8^) or similarly significant (the difference of *P* values less than 100 times) CDPs (Fig. 7d; Additional file 22). These results suggest that the dispensable sequences are an important part of the genome associated with phenotypic traits and underlying causes of missing heritability [58].

## Discussion

The dispensable sequences present in the population but absent from the reference assembly are important to understanding the genetic repertoire of a species [3]. The usual way to construct such non-reference sequences is assembling the deep-sequencing data of representative individuals [1, 7]. In this study, a metagenome-like assembly strategy was adopted to identify dispensable sequences by de novo assembly of low-coverage population sequencing data of 1483 rice accessions. Sequence heterozygosity, which was demonstrated as a major challenge facing this metagenome-like assembly strategy, was partly resolved by use of a large sample size to provide adequate sequencing depth for lots of dispensable sequences and categorizing the population into two groups to reduce the sequence diversity within the same group. Each dispensable sequence was assigned a genomic position relative to the Nipponbare reference genome. In addition, accurate sequences for different haplotypes of each dispensable sequence were constructed utilizing a local de novo reassembly strategy, which was shown to be an efficient solution to sequence heterozygosity. Various forms of evidence proved the feasibility and efficiency of these strategies. With the advance of next-generation sequencing and the availability of more and more population sequencing data [59], these strategies will become more useful for future studies in rice and other species.

We performed association mapping studies for agronomical and metabolic traits using dispensable sequences. GWASs have been fruitful in many species, especially in human. However, only SNPs that were identified against a reference genome have been used in nearly all studies. To the best of our knowledge, this is the first large-scale association mapping study using dispensable genomes. We found that 23.5 % of metabolic traits had higher association signals with polymorphisms of dispensable sequences than SNPs of the reference genome and 41.6 % of trait-associated SNPs had genomic locations concordant with associated dispensable sequences. These results suggest that the dispensable genome should be taken into account in association mapping and is an underlying cause of missing heritability. The dispensable sequences provide not only additional associated markers but also candidate genes, both of which are valuable resources for QTL mapping and GWASs.

Rice is an important crop that feeds half of the world population. Its yield and biotic/abiotic resistance need to be greatly improved in the coming decades to solve food shortages caused by population growth. In this study, more than 8000 protein-coding genes absent from the Nipponbare reference genome were successfully constructed. A proportion of proteins with annotated domains and families related to disease or stress resistance were found in the dispensable genome, such as LRR and NB-ARC domain-containing proteins, the stress-antifung family and WRKY family proteins. Together, these imply that the rice dispensable genome contains a significant amount of genetic resources that are useful for rice phenotype improvement.

Exploring the relationship between the dispensable genome and the reference genome is helpful for inspecting genome evolution. In this study, we observed 833 chromosome hotspots enriched with dispensable sequences. The formation mechanism of these hotspots needs further investigation. We also found 5146 contigs of the *indica* dispensable genome to be composed of exons and introns of different reference genes. Among 6302 annotated reference genes, the genes which encode enzymes and NBS-LRR domains were found to be significantly enriched compared with genes of the Nipponbare reference genome. This may indicate that parts of the dispensable sequences are formed by the shuffling of exons and introns of reference genes, which was reported to be involved in the formation of novel enzymes and NBS-LRR genes [60, 61]. Within the *indica* dispensable genome, 636 transposon clusters were found, more than 50 % of whose sequences contained at least two transposons. In addition, 8668 contigs were found to contain only one LTR or the internal region of a specific LTR retrotransposon and 896 contigs contained at least two of the three elements of a LTR retrotransposon. This indicates that the deletion of LTR retrotransposons, which was reported to be involved in the evolution of the rice genome, may contribute to the composition of the rice dispensable genome [2, 62]. All in all, our study, for the first time, has explored the dispensable genome of a higher organism at the population level. These findings will shed light on studies of new gene evolution, genome evolution and speciation.

## Conclusions

Our results suggest the feasibility and advantages of building the dispensable genome of a species using low-coverage population sequencing data. The sequences constructed will be helpful for understanding the rice dispensable genome and complementary to the reference genome for picking candidate genes associated with variations of agronomic and metabolic traits.

## Materials and methods

### Collection of sequence data

Low coverage (~1–3×) sequencing data of 1483 cultivated rice accessions, comprising 11.3 billion reads, were downloaded from the National Center for Biotechnology Information (NCBI) Sequence Read Archive (SRA) [GenBank: PRJNA171289] and European Nucleotide Archive [EMBL: ERP000106, ERP000729] (Additional file 1) [8].

### Genetic structure analysis of the rice population

We performed discriminant analysis of principal components based on 206,189 evenly distributed SNPs and classified the rice accessions into subgroups using the dapc function in the R package adegenet [63]. For SNP selection, we split the genome into ~5-kb regions; at most, three SNPs with minor allele frequencies ≥0.1 were randomly chosen for each region. The 1483 rice accessions were classified into four subgroups, *indica*, *aus*, temperate *japonica*, and tropical *japonica*, based on the results of population structure analysis and previous studies [8, 19]. Accessions of *indica* and *aus* groups were used to construct the *indica* dispensable genome while accessions of temperate *japonica* and tropical *japonica* groups were used to construct the *japonica* dispensable genome.

### Extraction of reads that could not be mapped to the reference genome

Both reads of a read pair of which at least one read could not be mapped to the Nipponbare reference genome (release 6.1 of the MSU Rice Genome Annotation Project) were collected to do de novo assembly. The mapped read is helpful for determining the relationship between the assembly results and the Nipponbare reference genome, and for inspecting the distribution of insertions in the Nipponbare genome. A stringent filtering rule was employed to remove low quality reads prior to assembly (Additional file 12). Jellyfish (version 1.1.5) [24] and Quake (version 0.3.0) [64] were used for computing *k*-mer frequencies and correcting sequencing errors in the reads before assembly (Additional file 12).

### The metagenome-like assembly strategy

Due to the low sequencing coverage of each accession, reads of various accessions were combined to do de novo assembly. The greatest challenge facing this approach is the substantial sequence variations between different accessions. To address this challenge, an assembly strategy used in metagenomic studies was introduced, since the mixed population sequencing data are similar to metagenomic sequencing data, which are a mixture of multiple species of a community. First, due to the substantial genomic differences between rice subspecies, all the 1483 accessions were categorized into two groups corresponding to either *indica* and *japonica* subspecies of rice and based on accession information and whole genome SNPs. Second, reads of each group were assembled independently using two assemblers, SOAPdenovo (version 1.05) [21] and Meta-IDBA (version 0.20, default parameters) [22] (Fig. 1a), which have been used widely in metagenomic studies [65]. SOAPdenovo (*k*-mer set as 63) was used with the specific parameter ‘-M 3’ as recommended for metagenomic data by a previous study [66]. Meta-IDBA is an iterative de Bruijn graph short read assembler specially designed for de novo metagenomic assembly. After discarding sequences shorter than 500 bp, the results of GICL were considered as the final assembly.

### Removal of contamination from the assembly result

Part of the reads which could not be mapped to the Nipponbare genome might be contamination. These sequences must be removed before further analysis. DeconSeq (version 0.4.1) [67] was applied in this step (Fig. 1a) in which several genome sequences were used to build the “remove” databases and the “retain” databases. The remove databases were used to screen for contaminations while the retain databases were used to eliminate redundant hits with higher similarity to non-contaminant genomes. Bacterial genomes, viral genomes, influenza genomes, and the human reference genome were all used to build the remove databases in this study while several plant genomes were used as the retain databases. Stringent parameters (5 % for coverage threshold, 90 % for identity threshold) were used for DeconSeq to filter potential contamination. All the genome sequences were downloaded from NCBI [68].

After the filtering by DeconSeq, the remaining sequences were further aligned to the NCBI Nucleotide collection database using Blastn (version 2.2.18) [44] to remove contamination. Sequences that were aligned to all sequenced plant genomes with matched lengths smaller than 300 bp were not subject to further analysis because of the difficulty of annotation, but these sequences are available for download from the Panrice website [16].

### PCR validation of de novo assembly

To evaluate the accuracy of the de novo assembly, PCR validation was performed using 30 contigs randomly selected from contigs with poor alignment (Blastn, query coverage <20 %) to any of the five sequenced genomes of the *Oryza* genus. PCR primers were designed to amplify DNA fragments with lengths ranging from 1162 bp to 2077 bp from these 30 randomly selected contigs (Additional file 5). For each contig, all the sequencing reads of different rice accessions were aligned to the contig using BWA (version 0.6.1) [20] and the genomic DNA of the rice accession with the highest read coverage for this contig was used as a template. For comparison, the genomic DNA of Nipponbare was also amplified using the designed primers of each contig.

### Sequenced genomes of the *Oryza* genus

Five sequenced genomes of the *Oryza* genus were used in this study: *Oryza sativa* L. ssp. *japonica* (Nipponbare) [69], *Oryza sativa* L. ssp. *indica* (*93-11*) [26, 70], *Oryza glaberrima* (African cultivated rice) [27, 70], *Oryza brachyantha* (African wild rice) [28, 70], and *Oryza rufipogon* (W1943, Asian wild rice) [71]. The genome sequences of *Oryza glaberrima*, *Oryza brachyantha*, and *Oryza rufipogon* were the results of the *Oryza* Map Alignment Project (OMAP), which aimed to generate reference sequences for collective *Oryza* genomes [72].

### Annotation of the assembly results

In spite of the successful construction of several cloned genes, the contig sequences obtained are expected to be error-prone. Annotation of these contigs is more troublesome than that of the usual genome. An integrative annotation pipeline was applied to predict protein-coding genes in the dispensable genome sequences (Additional file 12). Two ab initio gene predictors, Fgenesh (version 3.1.2) [39] and AUGUSTUS (version 2.5.5) [40], were used to predict protein-coding genes de novo. All the protein sequences of the grass family were downloaded from Gramene [70] and used as input to GeneWise (version 2.2.0) [41], which predicts protein-coding genes based on the homology between protein sequences and genome sequences. All the RNA-seq data of rice were downloaded from the NCBI SRA database and the website of the Comai Lab [73]. Low quality reads were filtered out by a stringent rule (Additional file 12) and the high quality reads were then mapped to the contigs using TopHat (version 2.0.4) [52]. Transcripts were further obtained from the mapping results using inchworm (version 2011-03-13) to do reference-guided assembly [74]. The assembled transcripts were then mapped to the contigs using PASA (version 2011-05-20) [42] and gmap (version 2012-04-05) [75]. Finally, the prediction results of Fgenesh, AUGUSTUS, and GeneWise and the alignment result of PASA were combined by EvidenceModeler (version 2012-06-25) [43], and were further used as input to PASA for updating. Predicted genes which could be aligned to the Nipponbare reference genome with ≥85 % coverage and ≥85 % identity were further removed to eliminate potential paralogs. The predicted genes that satisfied at least one of the following two rules were regarded as high confidence genes. First, more than 90 % of the coding region of a predicted gene is covered by protein sequences of the grass family and more than 60 % of the coding region is covered by any transcript assembled by inchworm. Second, the protein coded by a predicted gene can be aligned to a protein of the grass family with reciprocal coordinate overlap of more than 90 %.

To annotate transposable elements within the assembled contigs of the dispensable genomes, CENSOR (version 4.2.27) [54] was used taking the *Oryza* transposons in Repbase [76] as the repetitive library. The only transposons annotated were those with more than 80 % of their sequences aligned to a contig of the dispensable genome.

### Expression profiling of predicted genes of the dispensable genome

RNA sequencing data of ten experiments (Additional file 10) were collected from NCBI SRA database and the website of the Comai lab. These data were aligned to the dispensable genome independently using TopHat [52] with the predicted genes’ structures as input. The alignments were then used as input to Cufflinks (version 1.2.1) to assemble the reads into transcripts and quantify the expression level of each transcript [77].

### Identifying genomic positions relative to the Nipponbare genome from literature for genes absent from the Nipponbare genome

For *PSTOL1*, the bacterial artificial chromosome (BAC) sequence was reported to correspond to a 145-kb sequence interval between positions 15,321,347 and 15,466,417 on chromosome 12 of Nipponbare RefSeq (TIGR/MSU version 5). This genomic interval corresponds to positions between 15,321,347 and 15,466,417 on chromosome 12 of MSU version 6.1. The genomic position for *PSTOL1* was determined based on its position in the BAC sequence. Since the gene *Sub1B* was reported to be about 45 kb away from *Sub1A*, its sequence was aligned to the pseudomolecules of MSU version 6.1 and the resulting genomic interval was regarded as the genomic position of *Sub1A*. The BAC sequence harboring *Pikm1-TS* and *Pikm2-TS* was aligned to the pseudomolecules of MSU version 6.1 to get a genomic interval and the genomic positions of *Pikm1-TS* and *Pikm2-TS* were determined based on their positions in this BAC. The reported sequence [GenBank: AB433345] (11,150 bp) containing *GW5* of rice accession Kasalath was aligned to the pseudomolecules of MSU version 6.1 to get a genomic interval and the genomic position of *GW5* was determined based on its position in this sequence. The reported sequence [GenBank: AY986492] (2361 bp) containing *Xa27* of rice accession IRBB27 was aligned to the pseudomolecules of MSU version 6.1 to get a genomic interval and the genomic position of *Xa27* was determined based on its position in this sequence.

### Local de novo reassembly strategy

For a contig that can be assigned a genomic position relative to the Nipponbare genome, the distance between any two of the 1483 rice accessions was calculated using the 100 SNPs of the Nipponbare genome around this contig and accessions with a distance of zero between each other were grouped together. For a contig that could not be assigned a genomic position, all the 1483 rice accessions were divided into six groups based on the distance calculated using the whole genome SNPs (Additional file 23). Then the read pairs of each subgroup that could be mapped to this contig were assembled separately using Fuzzypath [78] (parameter “kmer” set as 31).

### Association mapping using the dispensable genomes

The sequencing depth of the dispensable sequences of each accession could be regarded as markers, which we termed “depth polymorphisms”. For each of the 533 rice accessions that we sequenced at ~2.5×, reads unmapped to the Nipponbare reference genome were aligned to both the *indica* and *japonica* dispensable genomes and the read depth at each position was obtained using SAMtools (samtools depth -q 10 -Q 20). Then the average read depth in 500-bp overlapping windows (with 250-bp step size) were computed for each rice accession. The average read depth larger than eight was arbitrarily set as eight. The depth values were further corrected by accounting for the average sequencing depth of each accession. We then transformed the continuous data (termed continuous DPs) into Boolean values (termed Boolean DPs) by setting out a threshold of 0.1 by manual inspection (values less than 0.1 were set to 0 and greater than 0.1 were set to 1). Only CDPs with the frequency of both 0 and 1 in BDPs ≥0.05 were used to carry out GWASs. We performed GWASs using methods described in our previous study [9], except that the FaST-LMM program was replaced by EMMAX [79], which is implemented in R from the MLMM package [80] and could deal with continuous values. The genetic and residual variance components were estimated by the restricted maximum likelihood (REML) method. The kinship coefficients (K matrix) used in linear mixed model were defined as the proportion of identical genotypes for 188,165 randomly sampled, evenly distributed SNPs for each pair of individuals, as were used in a previous study [9]. A total of 3,916,415 SNPs with minor allele frequency ≥0.05 that were identified by mapping reads to the reference genome in a previous study [9] for the same association panel were used to carry out GWASs. To save computing resources, only the most significant 1000 SNPs for each metabolic trait identified by FaST-LMM previously [9] were extracted and combined with CDP and BDP data to carry out GWASs using EMMAX. The genome-wide significance threshold (*P* = 6.6 × 10^−8^) used in a previous study for the same association panel was adopted [9].

## Additional files

Additional file 1: Table S1. Collection of sequence data. (DOCX 14 kb) Additional file 2: Table S2. Mapping rate of sequencing reads aligned to the Nipponbare genome of different accessions. (XLS 107 kb) Additional file 3:
**Supplementary figures.** (PDF 3352 kb) Additional file 4: Table S3. Alignment of contigs of the *indica* dispensable genome to different sequenced genomes of the *Oryza* genus. (XLS 8272 kb) Additional file 5: Table S4. PCR validation of 43 DNA fragments from 30 randomly selected contigs. *Primer position* is the position of a primer in the sequence of the contig. (XLS 19 kb) Additional file 6: Table S5. The alignment of genes in the sequenced region of Kasalath that harbors *PSTOL1* to the dispensable genome. (XLS 17 kb) Additional file 7: Table S6. Blastn alignment result of the full-length cDNA sequences of 12 O. *rufipogon* genes to the contigs of the dispensable genome. (DOC 35 kb) Additional file 8: Table S7. The alignment of predicted proteins of the *indica* dispensable genome to proteins coded by cloned genes of the Nipponbare genome using Blastp. (XLS 33 kb) Additional file 9: Table S8. The *Pfam* annotation results of the predicted proteins of the *indica* dispensable genome. See the Pfam website [81] for more details. (XLS 222 kb) Additional file 10: Table S9. RNA sequencing data used in the expression profiling of predicted genes of the dispensable genome. (XLS 10 kb) Additional file 11: Table S10. Sheet 1 shows the transposon annotation results of the *indica* dispensable genome given by Censor. Sheet 2 is the transposon annotation results of the *japonica* dispensable genome given by Censor. (XLS 5583 kb) Additional file 12:
**Supplementary methods.** (PDF 356 kb) Additional file 13: Table S11. Sheet 1 shows the genomic position relative to the Nipponbare genome for contigs of the *indica* dispensable genome. Sheet 2 shows the genomic position relative to the Nipponbare genome for contigs of the *japonica* dispensable genome. (XLS 22307 kb) Additional file 14: Table S12. The genomic positions relative to the Nipponbare genome of overlapping contigs of the *indica* and *japonica* dispensable genomes. Genomic positions were determined using an integration approach based on alignment and linkage disequilibrium for 7820 *indica* contigs corresponding to 7689 *japonica* contigs which were found to have reciprocal coordinate overlap of more than 60 % (identity ≥90 %). (XLS 1582 kb) Additional file 15: Table S13. Identified chromosome insertion hotspots. (XLS 51 kb) Additional file 16: Table S14. Sheet 1 shows contigs of the *indica* dispensable genome that were composed by reads mainly from a subgroup. Sheet 2 shows contigs of the *japonica* dispensable genome that were composed by reads mainly from a subgroup. Columns 2–4 show the percentage of reads mapped to the contig that belong to each subgroup. (XLS 1556 kb) Additional file 17: Table S15. Sheet 1 shows the contigs of the *indica* dispensable genome related to exon/intron shuffling. Sheet 2 displays the contigs of the *japonica* dispensable genome related to exon/intron shuffling. (XLS 3716 kb) Additional file 18: Table S16. Functional enrichment analysis of 6302 reference genes involved in the formation of non-reference sequences through exon/intron shuffling. The hmm accession for all the 6302 reference genes was extracted, and the number of genes with a specific hmm accession involved in the formation of non-reference sequences through exon/intron shuffling were compared with the whole genome level to find enriched accessions. In total, 3581 of these 6302 genes and 33,581 genes of the whole genome were annotated by Pfam. (DOC 40 kb) Additional file 19: Table S17. Sheet 1 shows the transposon clusters found within the *indica* dispensable genome. Sheet 2 shows the transposon clusters found within the *japonica* dispensable genome. The second to sixth columns show the percentage of query coverage of the alignment of the contig to different sequenced *Oryza* genome. (XLS 323 kb) Additional file 20: Table S18. LTR retrotransposons found within the dispensable genome. (XLS 2869 kb) Additional file 21: Table S19. Pack-MULEs found within the *indica* dispensable genome. (XLS 15 kb) Additional file 22: Table S20. Association mapping results of metabolic traits using whole genome SNPs or depth polymorphisms of the dispensable genome. *Lead_SNP* is the SNP with the most significant association signal for a specific metabolic trait. *Chromosome* is the chromosome position of Lead_SNP. *Position* is the chromosome position of Lead_SNP. *P value* is the association *P* value for Lead_SNP. *Close_DP* is the associated depth polymorphism closest to Lead_SNP. *Close_DP_P value* is the association *P* value for close_DP. *Close_DP_Position* is the genomic position of Close_DP relative to the Nipponbare genome. *Close_DP_Distance* is the distance (kb) between the Lead_SNP and the Close_DP. *Lead_CDP* is the continuous depth polymorphism with the most significant association signal for a specific metabolic trait. *Lead_CDP_P value* is the association *P* value for Lead_CDP. *Lead_CDP_Pfam* is the Pfam prediction result for Lead_CDP. *Lead_BDP* is the Boolean depth polymorphism with the most significant association signal for a specific metabolic trait. *Lead_BDP_P value* is the association *P* value for Lead_BDP. *Lead_BDP_Pfam* is the Pfam prediction result for Lead_BDP. (XLS 496 kb) Additional file 23: Table S21. The rice accessions were classified into six groups (groups A–F) to do local reassembly for contigs which could not be assigned genomic positions relative to the reference genome. (XLS 86 kb)

## Abbreviations

BAC: bacterial artificial chromosome
BDP: Boolean depth polymorphism
bp: base pair
CDP: continuous depth polymorphism
DP: depth polymorphism
GWAS: genome-wide association study
LD: linkage disequilibrium
LMM: Linear mixed model
LRR: leucine-rich repeat
LTR: long terminal repeat
MITE: miniature inverted-repeat transposable element
MSU: Michigan State University
MULE: *Mutator*-like transposable element
NCBI: National Center for Biotechnology Information
PCR: polymerase chain reaction
QTL: quantitative trait locus
RPKM: reads/per kilobase merged exonic region/per million mapped reads
SNP: single nucleotide polymorphism
SRA: Sequence Read Archive.
