# Supplementary material for: Exploring the rice dispensable genome using a metagenome-like assembly strategy
Source: Genome Biol. 2015 Sep 7;16:187. doi: 10.1186/s13059-015-0757-3 (PMC4583175; doi:10.1186/s13059-015-0757-3)
Supplement: Additional file 1: Table S1. — Collection of sequence data. (DOCX 14 kb) [file 13059_2015_757_MOESM1_ESM.docx]

**Additional file 1: Table 1. Collection of sequence data.**

| Accession Number in EBI or NCBI | Description | Read Count | Base Count | References |
| --- | --- | --- | --- | --- |
| PRJNA171289 | Resequencing of 533 rice accessions consisted of both landraces and elite varieties | 3,364,045,886 | 605,485,409,801 | Chen et al., 2014 |
| ERP000106 | Resequencing of 620 rice genomes for genome-wide association studies | 1,607,895,970 | 234,752,811,620 | Huang et al., 2010; Huang et al., 2011; Huang et al., 2012 |
| ERP000729^a^ | Resequencing of 462 rice accessions from world-wide germplasm collection | 480,530,066 | 70,157,389,636 | Huang et al., 2011; Huang et al., 2012 |

^a^ Only the data of 330 rice accessions released in Huang et al., 2011 were used.
