# Supplementary material for: Exploring the rice dispensable genome using a metagenome-like assembly strategy
Source: Genome Biol. 2015 Sep 7;16:187. doi: 10.1186/s13059-015-0757-3 (PMC4583175; doi:10.1186/s13059-015-0757-3)
Supplement: Additional file 3: — Supplementary figures. (PDF 3352 kb) [file 13059_2015_757_MOESM3_ESM.pdf]

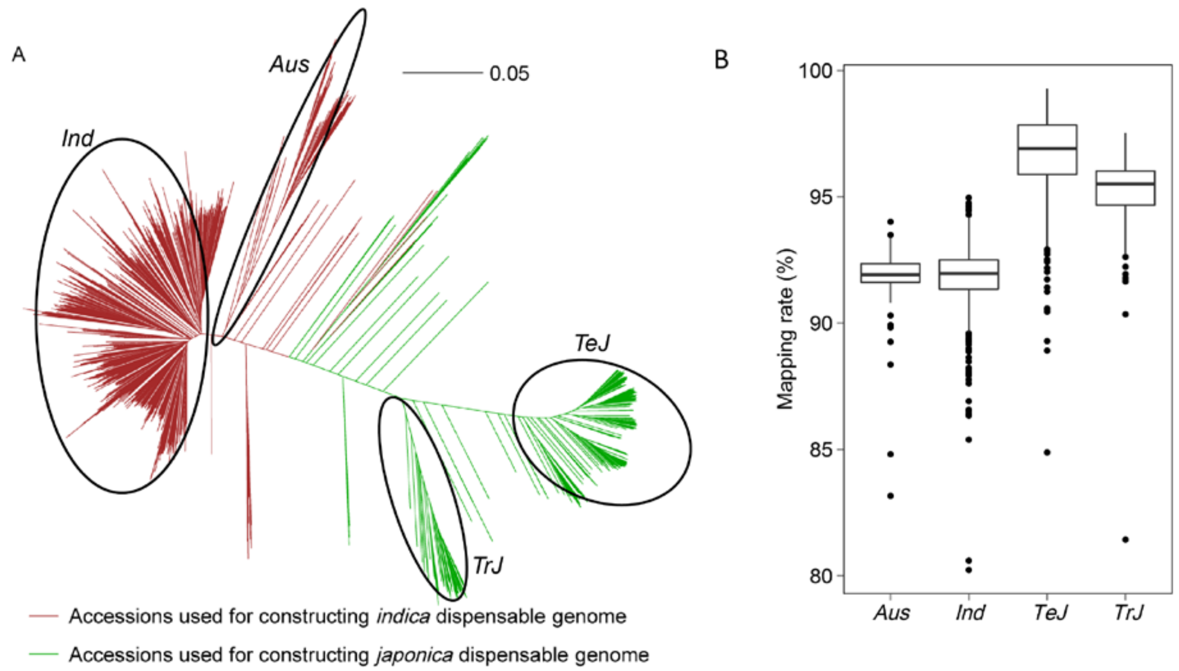

Additional file 3: Figure S1. The classification of rice accessions used to construct the *indica* and *japonica* dispensable genomes and the mapping rates of different subpopulations.

(A) Neighbor-joining tree of 1,483 accessions. The 1,483 rice accessions were classified into four subgroups, *indica* (Ind), *aus* (Aus), temperate *japonica* (TeJ) and tropical *japonica* (TrJ) based on previous study and results of population structure analysis. Accessions of the *Ind* and *Aus* group were used to construct the *indica* dispensable genome while accessions of the *TeJ* and *TrJ* group were used to construct the *japonica* dispensable genome. (B) The mapping rates of all the accessions in each subgroup were shown as different boxplots. Accessions with mapping rates lower than 80% were filtered out in advance.

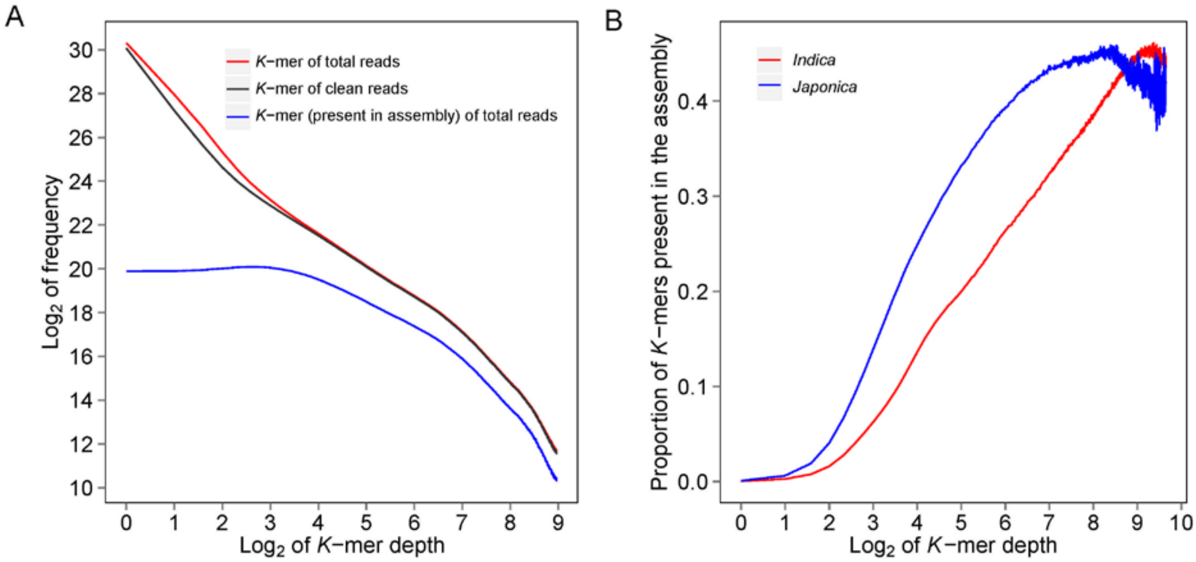

Additional file 3: Figure S2. Evaluation of the assembly strategy by investigating *k*-mer distributions of reads of the *japonica* accessions.

(A) The number of *k*-mers with different depth in total sequencing reads and clean reads. (B) The proportion of *k*-mers present in the assembly for *k*-mers with various depth in all sequencing reads. red, reads used to build the *indica* dispensable genome. blue, reads used to build the *japonica* dispensable genome.

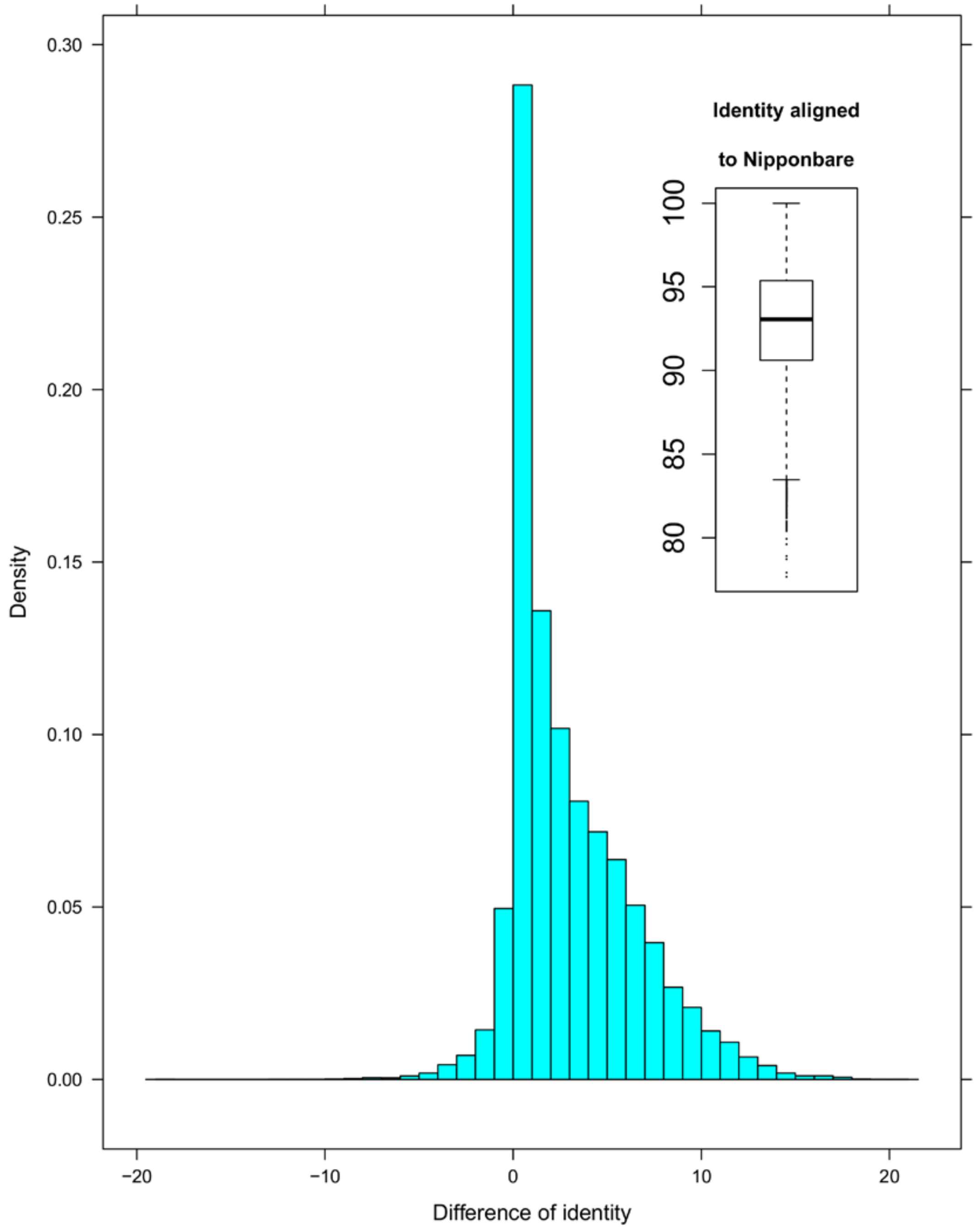

Additional file 3: Figure S3. The difference of alignment identities of 17,956 contigs aligned to non-reference genome and the Nipponbare genome.

These 17,956 contigs were assigned a non-reference genome based on the alignment score when aligned to different sequenced genomes of the *Oryza* genus. The histogram shows the distribution of the identity of these 17,956 contigs aligned to non-reference genome minus the identity aligned to the Nipponbare genome. The boxplot shows the identity of these 17,956 contigs aligned to the Nipponbare genome.

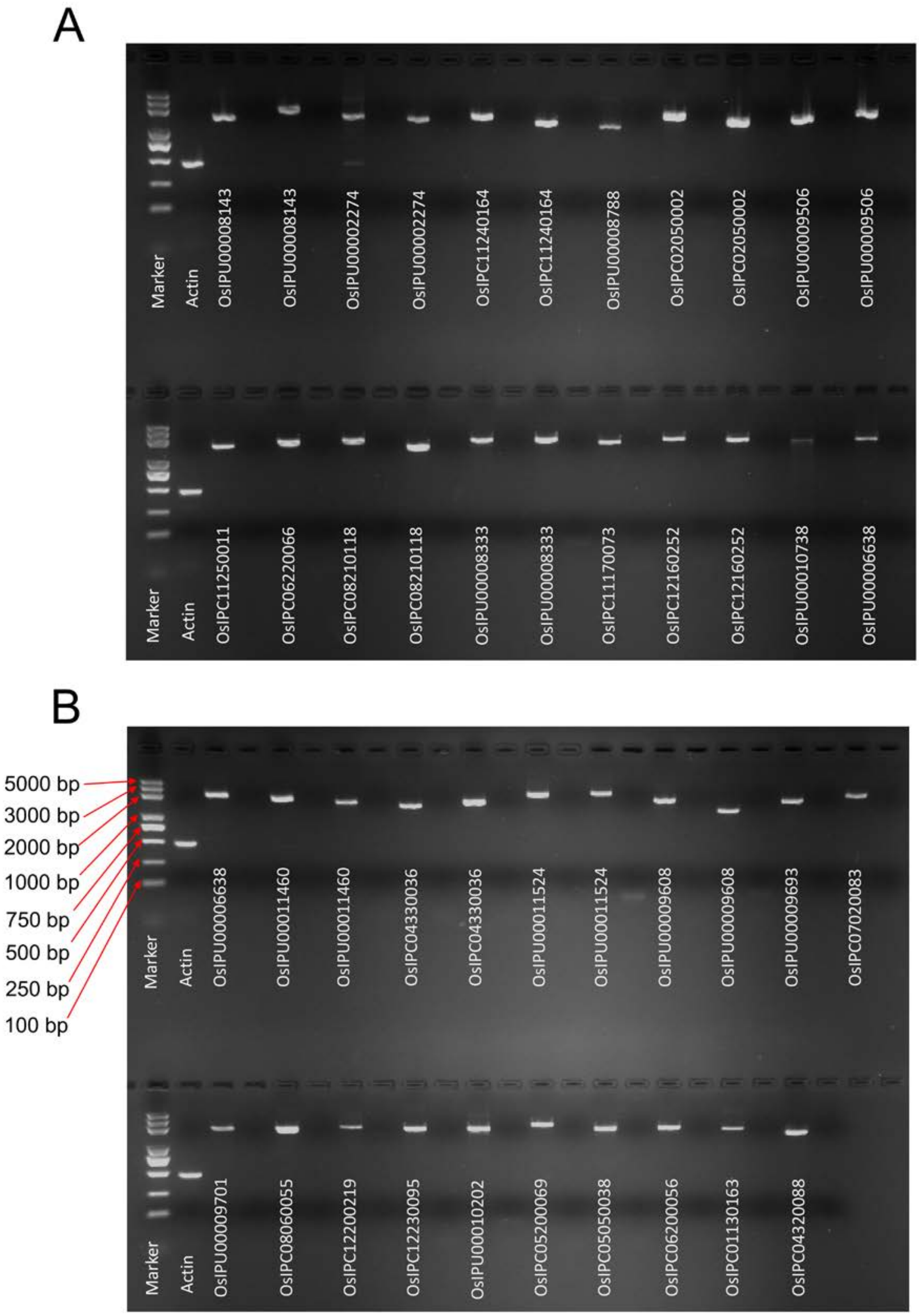

Additional file 3: Figure S4. PCR validation of 43 DNA fragments from 30 randomly selected contigs of the *indica* dispensable genome.

For each DNA fragment, 2 PCR results are shown. The lanes marked with contig names are PCR results using DNA of selected rice accessions while the unmarked lanes are PCR results

using DNA of Nipponbare (See Supplementary Table 4 for more details). Lanes marked with "Actin" are the PCR results of actin using DNA of Nipponbare. The amplification size of markers fragments are indicated in B.

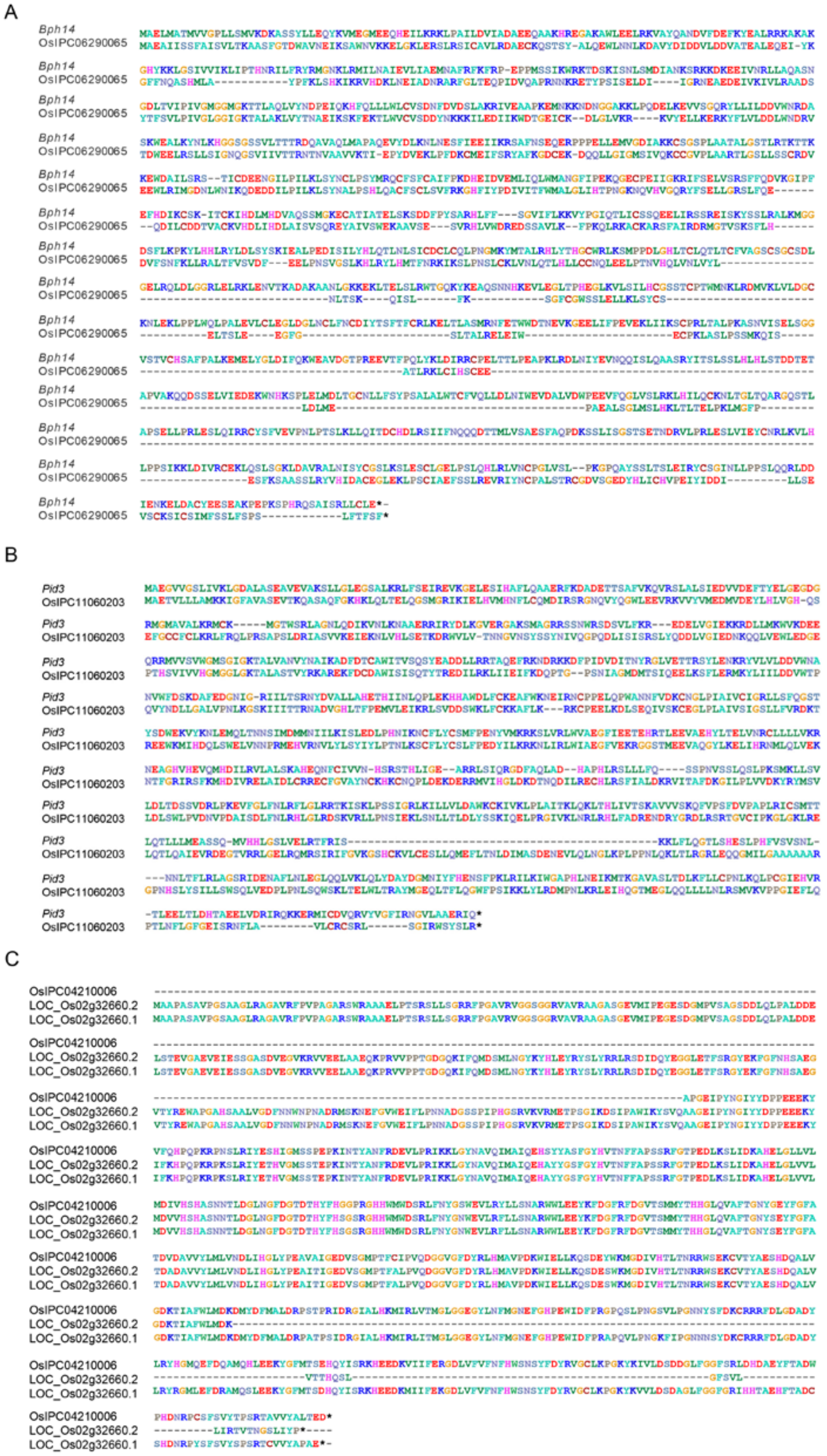

Additional file 3: Figure S5. The alignment of protein sequences of the *indica* dispensable genome and the encoded protein of the cloned genes *Bph14*, *Pid3* and *OsBEIIb*.

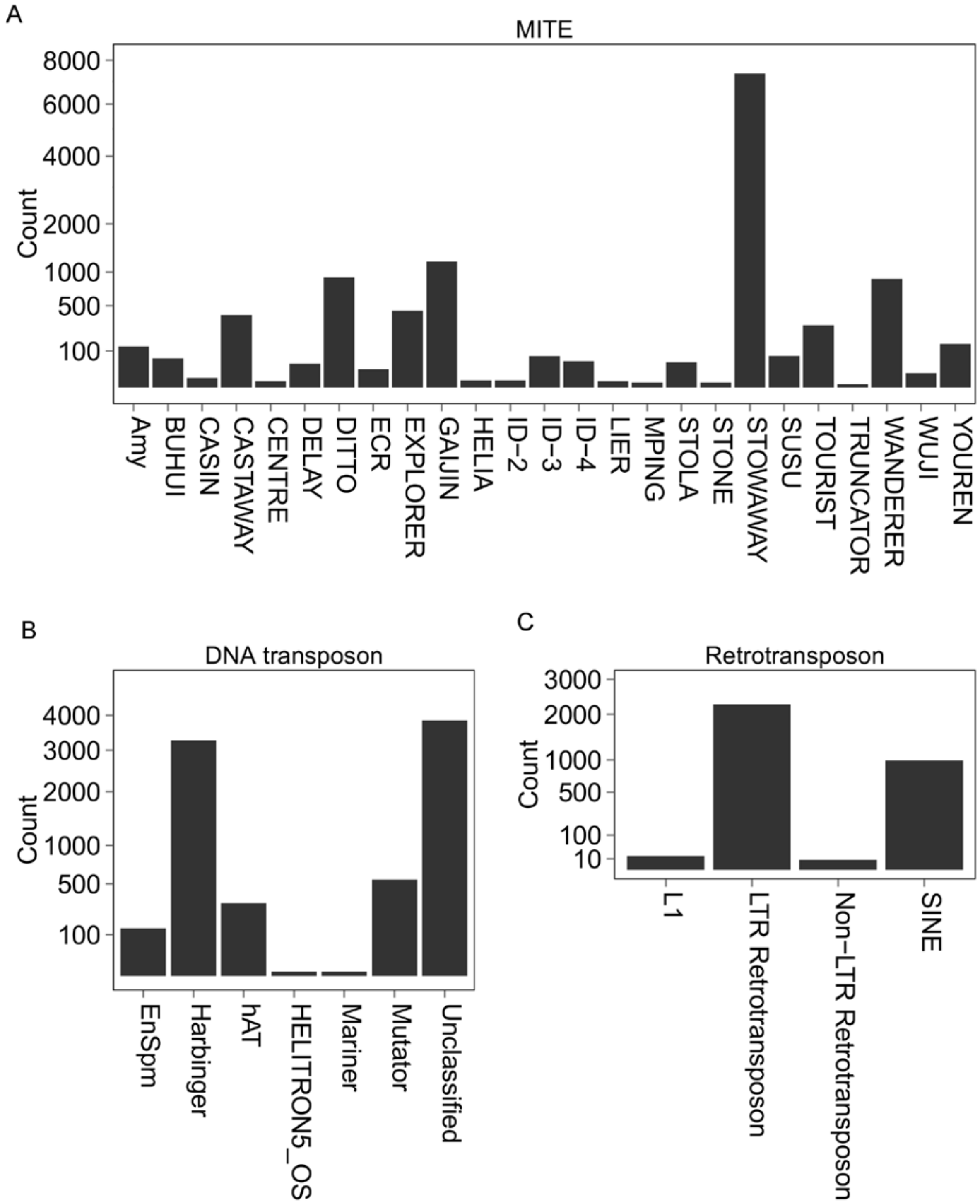

Additional file 3: Figure S6. The transposon composition of the *indica* dispensable genome.

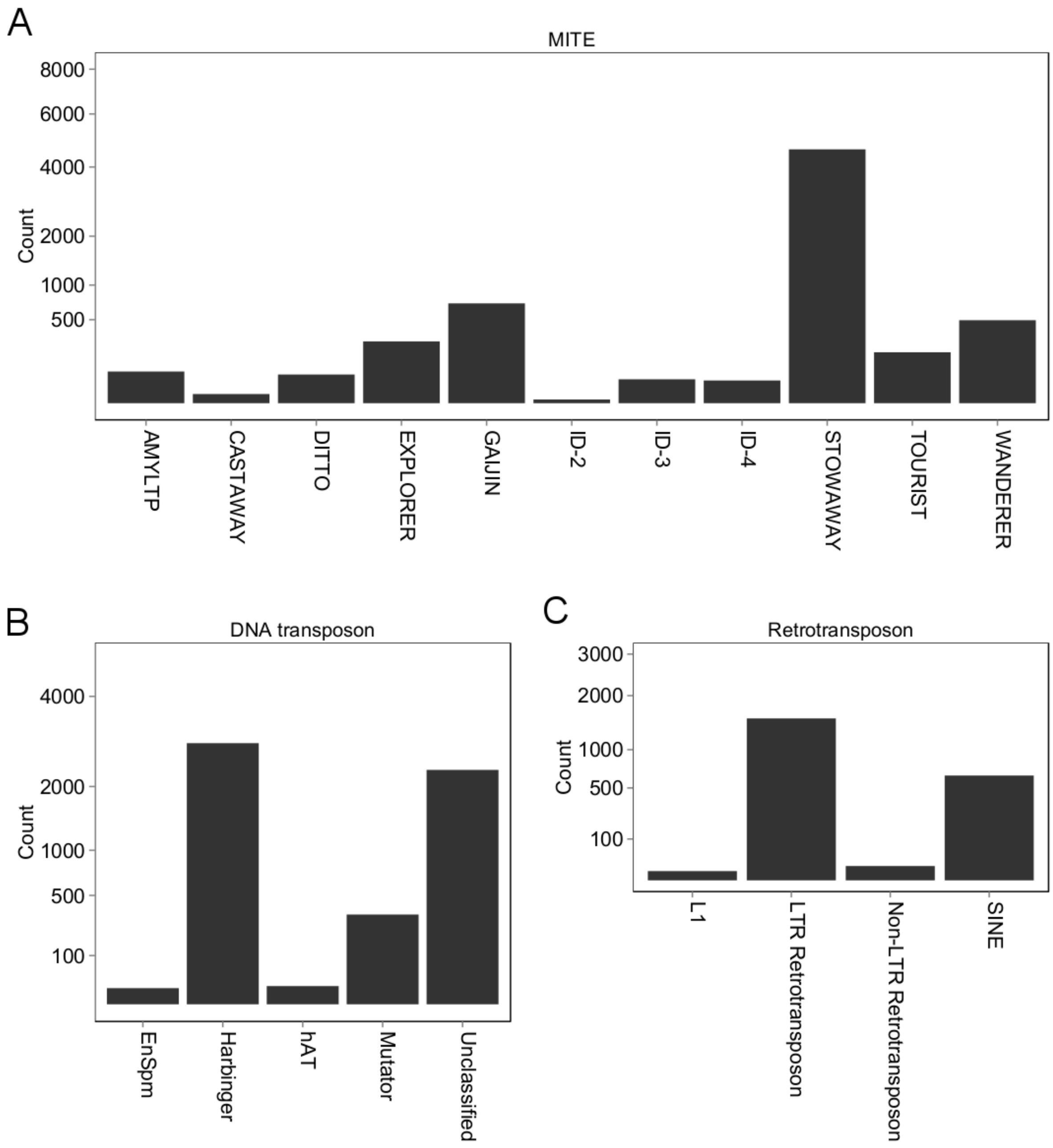

Additional file 3: Figure S7. The transposon composition of the *japonica* dispensable genome.

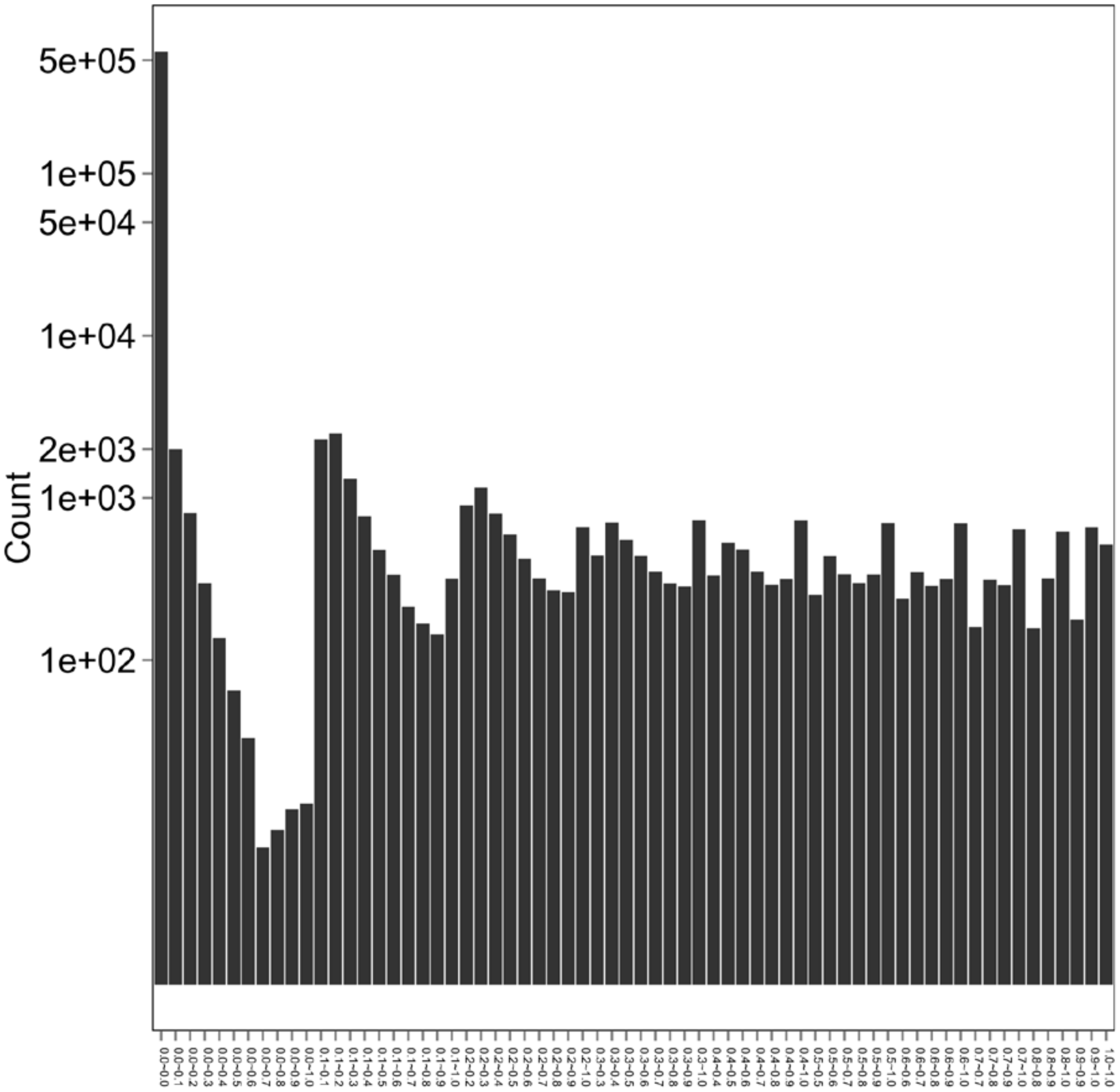

Additional file 3: Figure S8. The bar plot of *indica-japonica* contig pairs that were located within nearby regions (distance ≤50 kb) with specific match length divided by the length of the *indica* and *japonica* contigs respectively.

The numbers on the *x*-axis signify the match length between *indica-japonica* contig pairs divided by the length of the *indica* and *japonica* contigs.

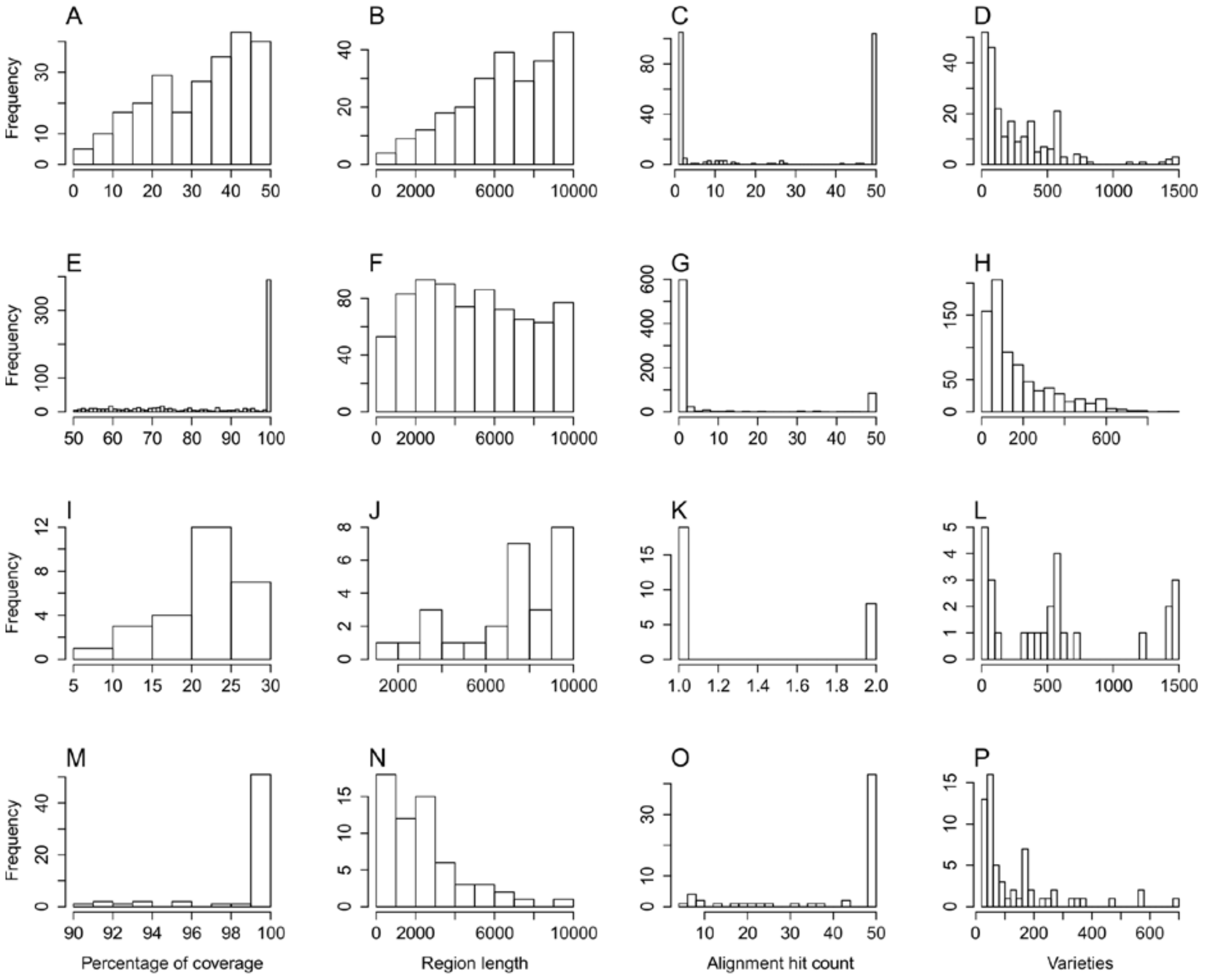

Additional file 3: Figure S9. Evaluation of local assembly strategy with simulation data. (A-D) show 243 genomic regions with poor local assembly results (type I). (E-H) show 756 regions with pretty good local assembly results (type II). (I-L) show 27 type I regions that were in the unique part of the genome. (M-P) show 61 type II regions in the repeat part of the genome. (A) (E) (I) (M) show the percentage of coverage of the alignment of the local assembly to the original sequence of the selected genomic region. (B) (F) (J) (N) show the lengths of the selected regions. (C) (G) (K) (O) show distributions of the number of alignment hits to the whole genome for the selected region. The number of alignment hits larger than 50 was set as 50. (D) (H) (L) (P) show the number of accessions used to construct the longest local assembly.

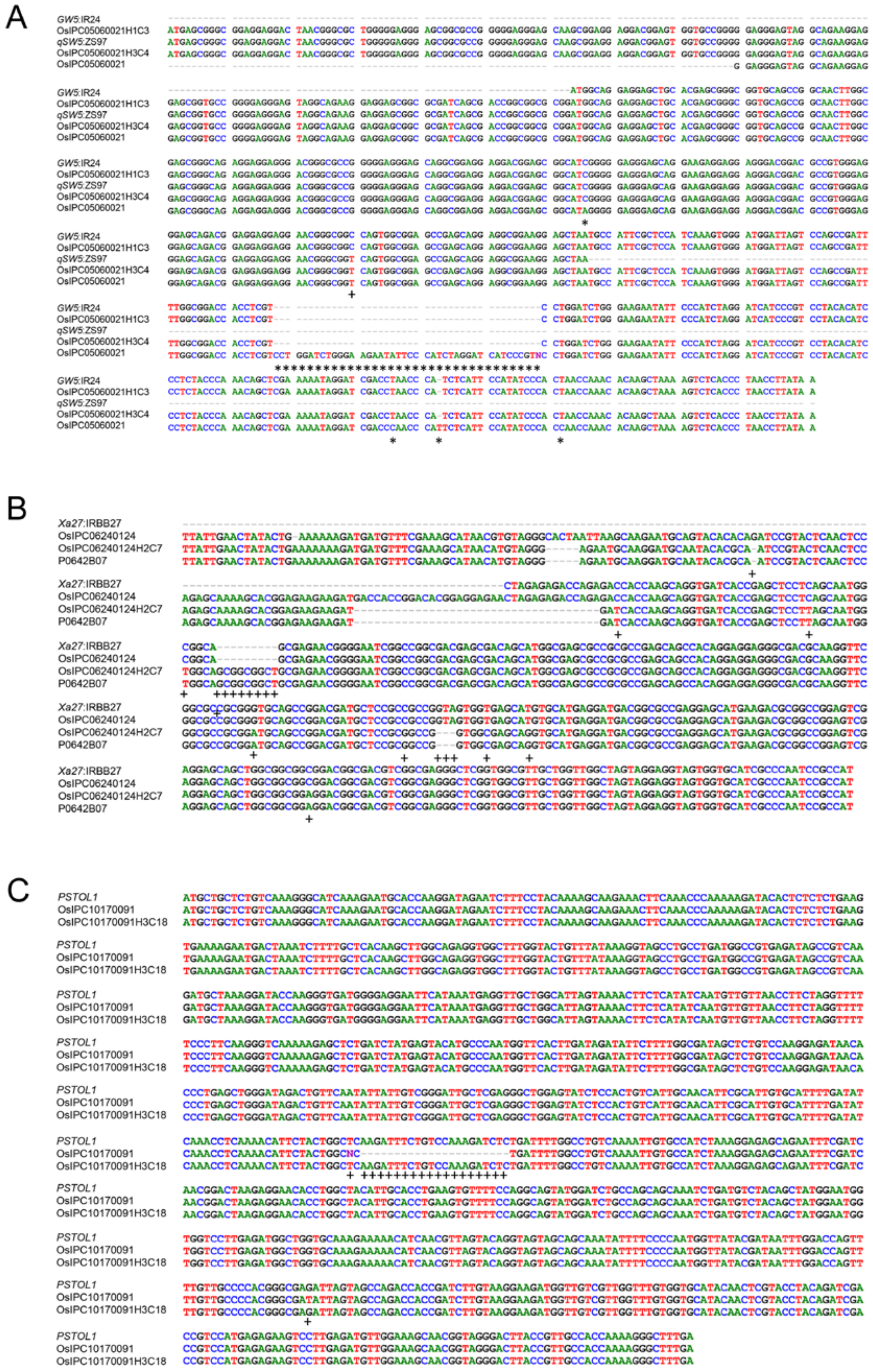
A
GW5:IR24
OsIPC05060021H1C3
qSW5:ZS97
OsIPC05060021H3C4
OsIPC05060021
B
Xa27:IRBB27
OsIPC06240124
OsIPC06240124H2C7
P0642B07
C
PSTOL1
OsIPC10170091
OsIPC10170091H3C18

Additional file 3: Figure S10. The local de novo reassembly results for the contig harboring *GW5*, the contig harboring *Xa27* and the contig harboring *PSTOL1*.

(A) *GW5:IR24* and *qSW5:ZS97* represent two haplotypes of *GW5*. OsIPC05060021 is the raw assembly result for *GW5* and OsIPC05060021H1C3 and OsIPC05060021H3C4 are the local reassembly results of two different haplotypes. The asterisk marks the errors introduced by the raw assembly and the plus sign (+) indicates the SNP between the two haplotypes of *GW5*. (B) *Xa27-IRBB27*, the original sequence of *Xa27*. OsIPC06240124 is the raw assembly of the contig harboring *Xa27*. OsIPC06240124H2C7 is the local *de novo* reassembly results for the contig harboring *Xa27*. P0642B07 is a haplotype of *Xa27*. (C) *PSTOL1*, the original sequence of *PSTOL1*. OsIPC10170091 is the raw assembly of the contig harboring *PSTOL1*. OsIPC10170091H3C18 is the local *de novo* reassembly results for the contig harboring *PSTOL1*.

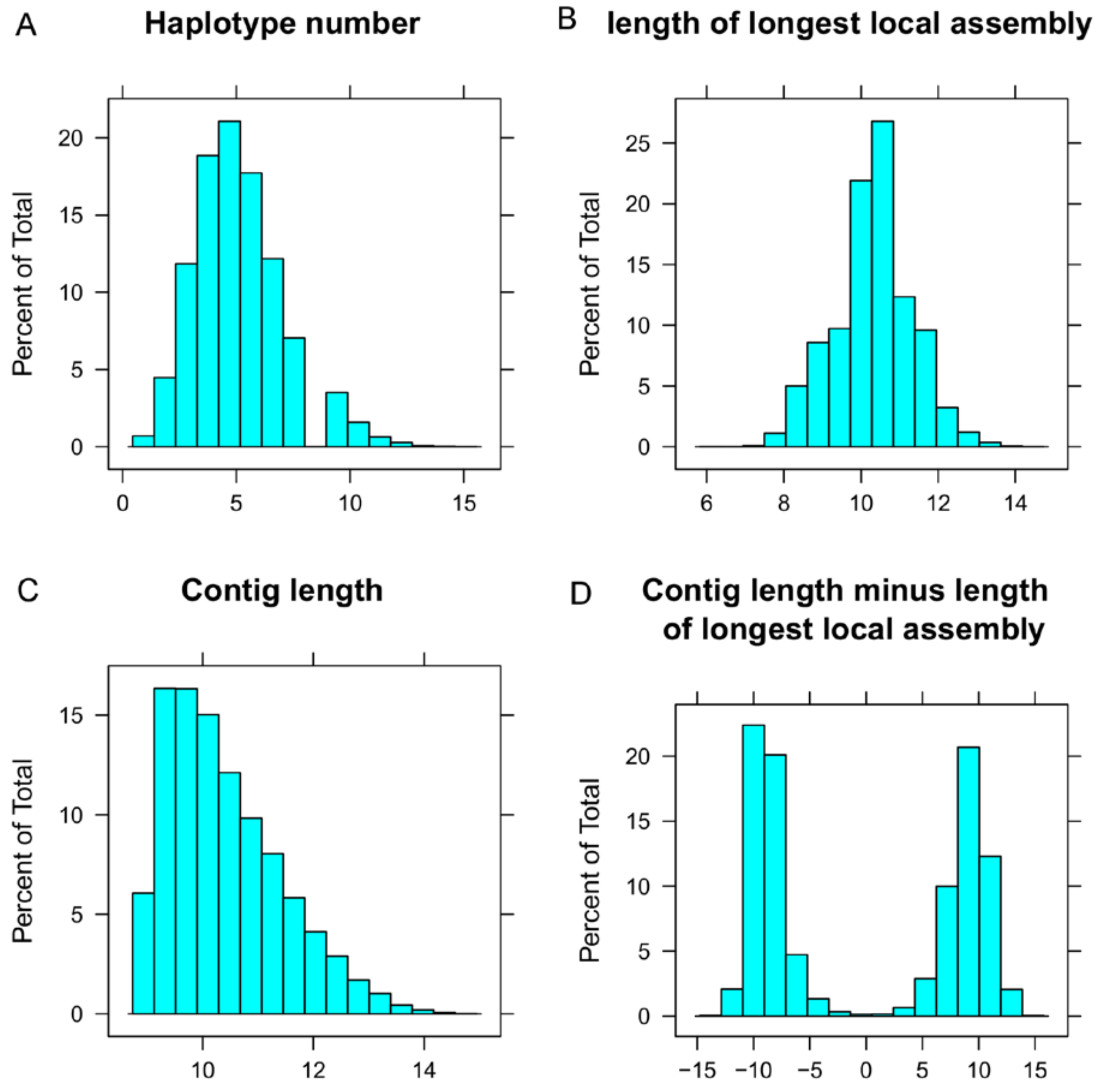

Additional file 3: Figure S11. The local de novo reassembly results of the *indica* dispensable genome.

(A) The distribution of haplotype numbers of each contig determined by local SNPs. Haplotypes with too few accessions (≤50) were filtered out. (B) The distribution of the length of the longest local assembly of the *indica* dispensable genome. (C) The distribution of the lengths of contigs of the *indica* dispensable genome (in log2 ratio). (D) The distribution of the contig length minus the length of the longest local assembly (in log2 ratio).

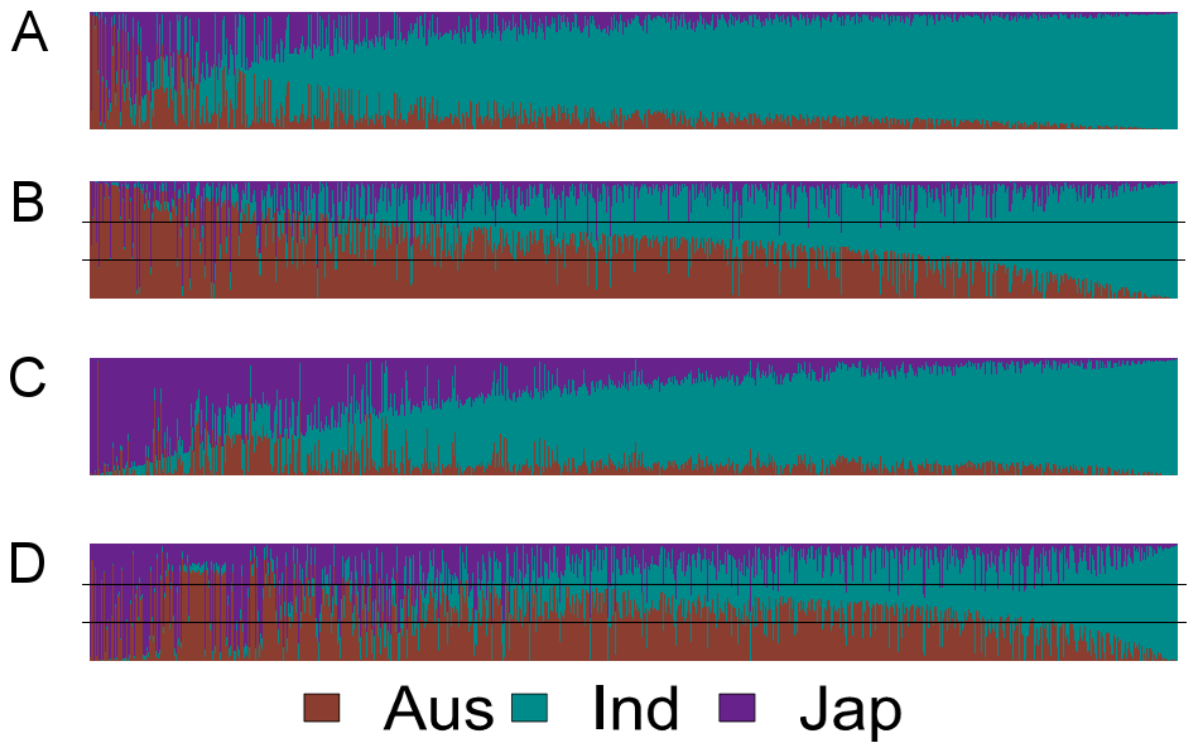

Additional file 3: Figure S12. Population composition of contigs of the dispensable genome. Each contig is represented by a vertical bar and the length of each colored segment in each vertical bar represents the proportion contributed by different populations. (A) is the unweighted population composition of contigs of the *indica* dispensable genome. (B) is the weighted population composition of contigs of the *indica* dispensable genome. (C) is the unweighted population composition of contigs of the *japonica* dispensable genome. (D) is the weighted population composition of contigs of the *japonica* dispensable genome. *Ind*: *Indica*, *Jap*: *Japonica*. The horizontal lines in B and D signify the values of 0.33 and 0.66.

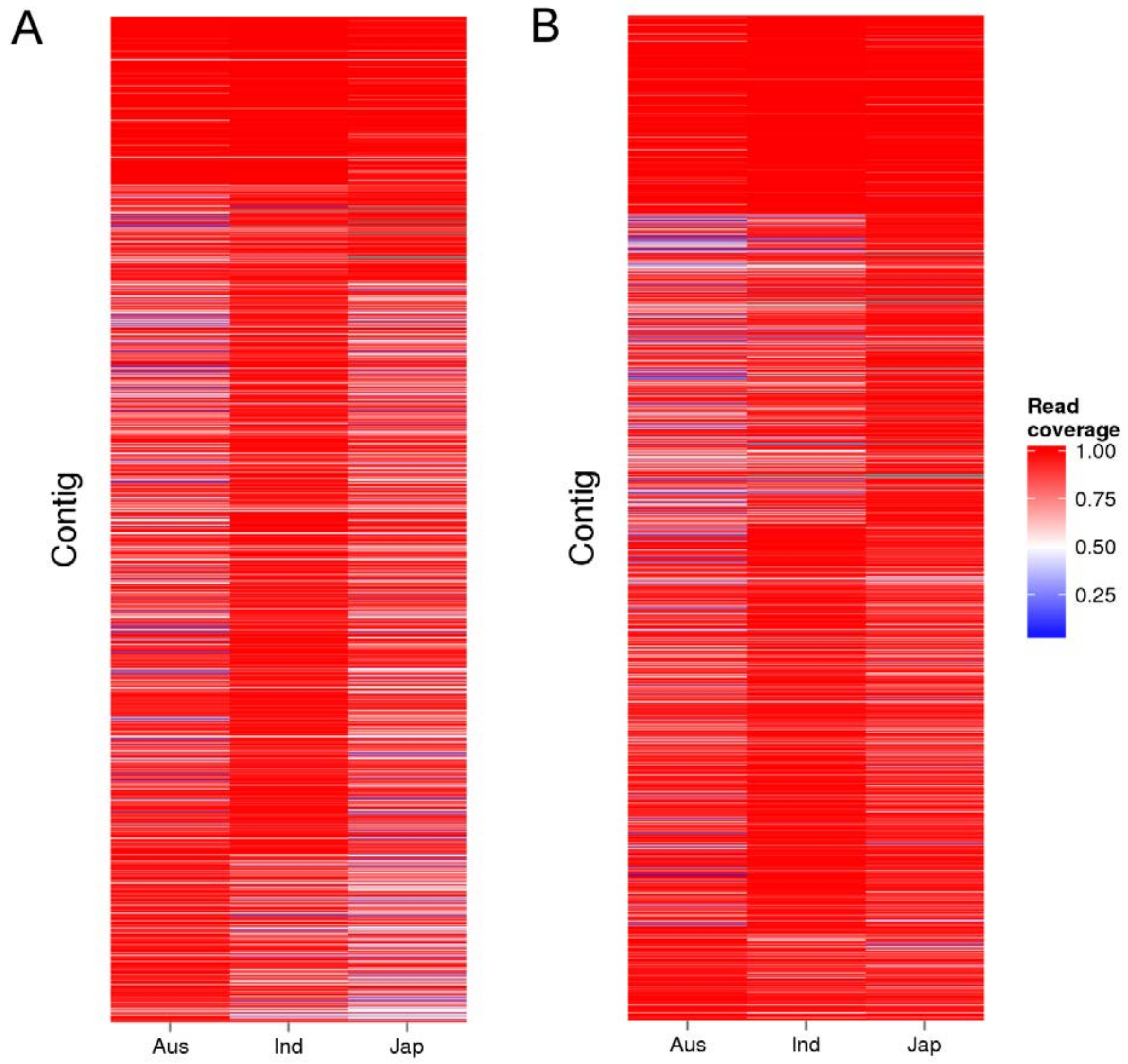

Additional file 3: Figure S13. Read coverage of the contigs of the dispensable genome. The 1,483 rice accessions used in this study were classified into 5 subgroups based on whole genome SNP (See supplementary Table 2). The read coverage of a contig for each rice accession was calculated based on the alignment of raw reads of this accession to the sequence of this contig. The read coverage of a contig for a subgroup was represented by the read coverage of the accession with the highest read coverage within this subgroup (Only three subgroups were shown). (A) 52,972 contigs of the *indica* dispensable genome. (B) 30,349 contigs of the *japonica* dispensable genome.

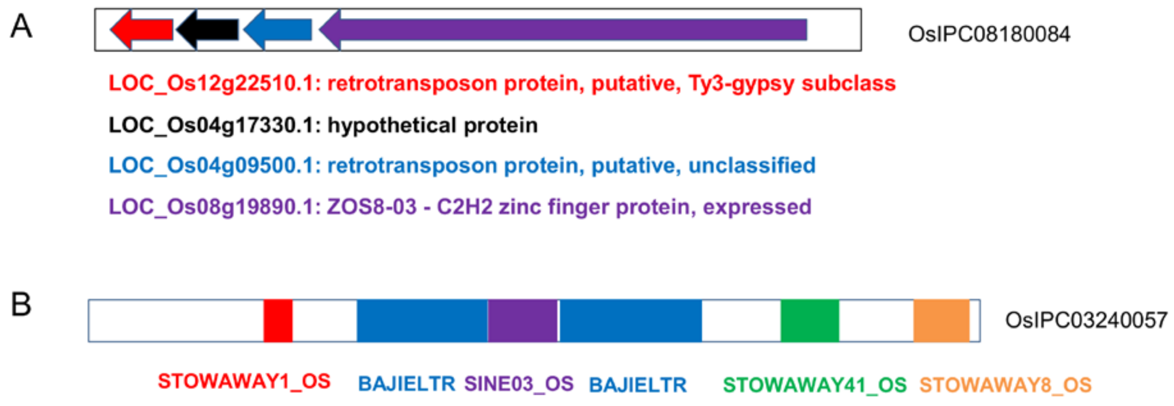

Additional file 3: Figure S14. The composition of dispensable sequences.

(A) The contig (OsIPC08180084) is composed of exons of four different reference genes represented as four arrows. The four exons and the annotation of corresponding genes are indicated in the same color. (B) The transposon composition of a contig (OsIPC03240057) of the *indica* dispensable genome.

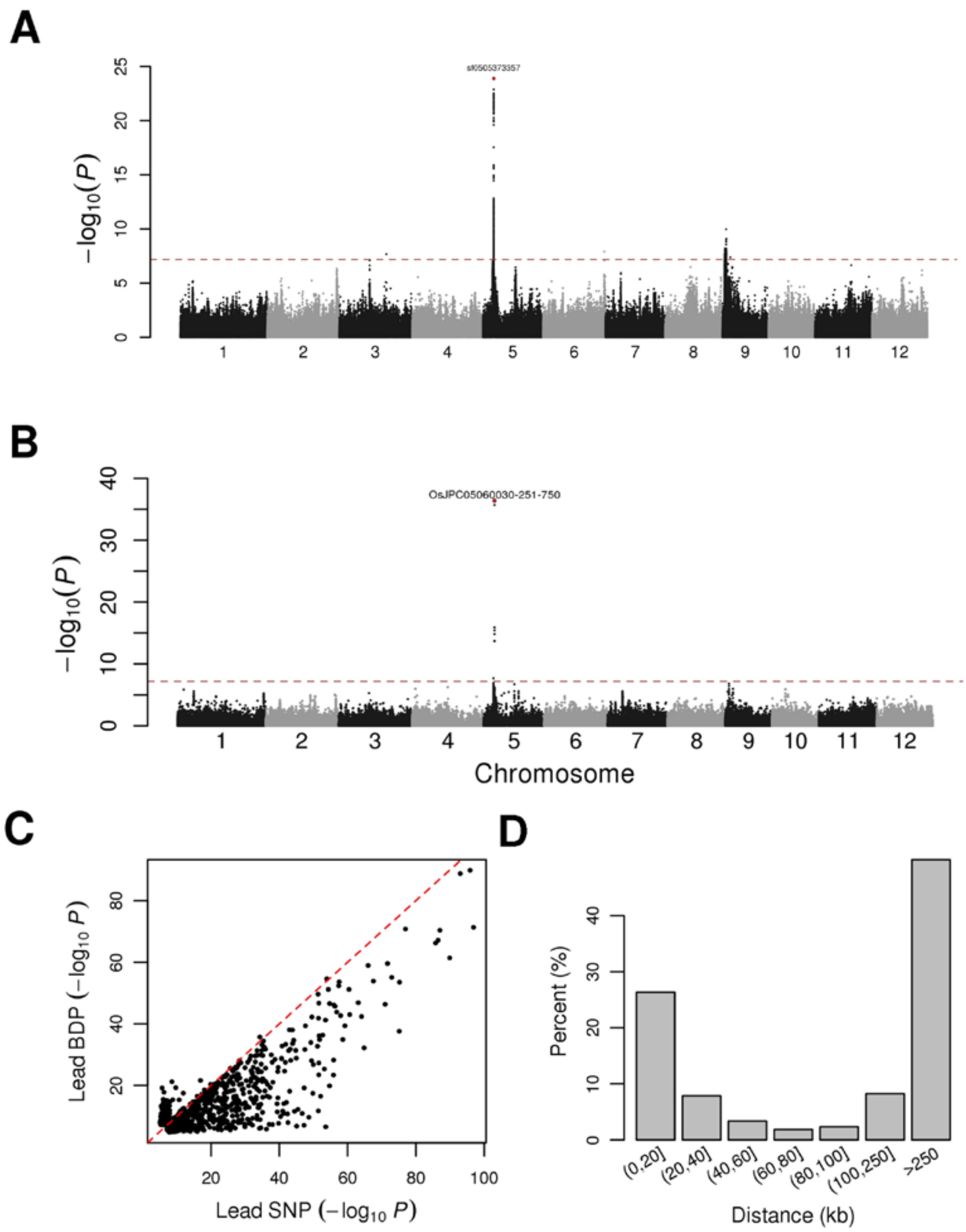

Additional file 3: Figure S15. Comparisons of association mapping using LMM based on reference and dispensable genomes.

(A) Manhattan plots for association mapping of rice grain width using SNPs based on the reference genome. (B) Manhattan plots for association mapping of rice grain width using BDPs based on the dispensable genomes. The horizontal dashed line indicates the genome-wide significance threshold ($P = 6.6\times10^{-8}$). The lead BDP was marked in brown and labeled ($P = 4.0\times10^{-37}$). (C) Comparisons of LMM *P*-values of lead SNPs with that of lead BDPs for 1,169 metabolic traits of which the *P*-values for the lead SNPs or the lead BDPs

passed the significance threshold. (D) The distribution of chromosome distances between significant lead SNPs and the closest significant ($P \leq 6.6\times10^{-8}$) or similarly significant (the difference of P-values less than hundred times) BDPs.
