# Supplementary material for: Exploring the rice dispensable genome using a metagenome-like assembly strategy
Source: Genome Biol. 2015 Sep 7;16:187. doi: 10.1186/s13059-015-0757-3 (PMC4583175; doi:10.1186/s13059-015-0757-3)
Supplement: Additional file 7: Table S6. — Blastn alignment result of the full-length cDNA sequences of 12 O. rufipogon genes to the contigs of the dispensable genome. (DOC 35 kb) [file 13059_2015_757_MOESM7_ESM.doc]

**Additional file 7: Table S6: Blastn alignment result of the full-length cDNA sequences of 12 O. *rufipogon* genes to the contigs of the dispensable genome.**

| **Query** | **Subject** | **Query coverage** | **Identity** |
| --- | --- | --- | --- |
| CT841704 | OsIPC01270012 | 65% | 92% |
| CT841911 | OsIPC01270012 | 54% | 91% |
| CT841912 | OsJPU00005881 | 100% | 100% |
| CT842002 | OsIPC03180054 | 97% | 99% |
| CU405720 | OsJPC04020127 | 99% | 100% |
| CU405911 | OsJPC10170015 | 73% | 89% |
| CU406202 | OsIPU00010795 | 100% | 99% |
| CU406257 | OsIPC11280438 | 100% | 99% |
| CU406339 | OsIPC03070012H4C4 | 83% | 99% |
| CU406702 | OsIPC12080081 | 98% | 89% |
| CU406871 | OsIPC09050034 | 98% | 94% |
| CU406924 | OsIPC09080142 | 100% | 98% |
