# Supplementary material for: Exploring the rice dispensable genome using a metagenome-like assembly strategy
Source: Genome Biol. 2015 Sep 7;16:187. doi: 10.1186/s13059-015-0757-3 (PMC4583175; doi:10.1186/s13059-015-0757-3)
Supplement: Additional file 12: — Supplementary methods. (PDF 356 kb) [file 13059_2015_757_MOESM12_ESM.pdf]

Additional file 12

1.1. Filtering low quality read pairs and read correction prior to assembly

Low quality reads are pernicious to the sequence assembly process and should be removed prior to assembly. If the quality of more than 15% of the bases of a read pair is lower than 5 or the quality of more than 50% of the bases of a read pair is lower than 15, this read pair would be filtered out. The reads of all accessions, *indica* and *japonica* separately, were mixed to count *K*-mer occurrences by Jellyfish [1] (*k*-mer size set to be 19 bp). The counting results were then used as input to Quake [2] to correct sequencing errors. The coverage cutoff for Quake was set to 1 as was recommended by the author.

1.2. Sequence annotation for assembled contigs

Fgenesh [3] was used with the parameter "organism" set to be "monocot plants". The parameter "species" for Augustus [4] was set as "maize" and only complete gene models were predicted. All the protein sequences of the grass family were aligned to the contigs of the dispensable genome by Blastp [5] (e-value cutoff 1e-10). For a specific contig, only the protein sequences that could be aligned to the contig with query coverage higher than 50% of the length of the protein were used as input to Genewise [6] for annotation.

For all the RNA-seq data downloaded from NCBI SRA database, low quality (≤5) bases at the 3' end were trimmed first. Reads shorter than 35 bp after trimming were discarded. For a read passing this filtering, if the quality of more than 15% of its bases were lower than 5 or the quality of more than 50% of its bases were lower than 15, this read would be filtered out.

1.3. Integration of alignment results and LD based approach to place each contig to the rice chromosome

Utilizing the LD based approach, each SNP of a contig determined a chromosome location of this contig. The vote for each location of a contig was calculated by measuring each location in 100 kb unit. Two chromosome locations with the highest and the second highest votes were

recorded. If the two locations were less than 1 Mb away from each other, they were considered as one location taking the location with the highest vote as the final location. If the highest vote was three or more fold as much as the second highest vote, the location with the second highest vote would be abandoned.

Each contig was aligned to the Nipponbare genome using Blastn [5] and hits with the top three highest scores were retained.

For a contig with the locations determined only by the LD based approach, if there were two locations determined by LD based approach or the highest vote was smaller than 3, the location of this contig could not be determined. Otherwise, the location with the highest vote would be assigned to this contig.

For a contig with the locations determined only by the alignment approach, if the score of the best hit was not 5 or more fold as much as the second best hit, the location of this contig could not be determined. Otherwise, the location of the best alignment hit was assigned to this contig.

For a contig with the locations determined by both the LD based approach and the alignment approach, the chromosome distances between the locations determined by LD based approach and the locations determined by alignment approach were calculated. If the nearest distance was smaller than 1 Mb, the location determined by the alignment approach of this nearest distance was considered as the location of this contig. Otherwise, the location assigned by the LD based approach would be taken as the location of this contig, if there was only one location assigned by the LD based approach and the vote for this location was larger than 3.

1.4. Identification of chromosome insertion hotspots

The Nipponbare chromosomes were split into non-overlapping 10-kb windows and the number of hanging read pairs in each window were counted. Windows in which the number of hanging read pairs exceeded 3493 (the 90th quantile of all windows) were collected for further inspection by counting the number of contigs of the dispensable genome located in each window. Windows in which the number of contigs were more than 7 (the 90th quantile of

all the windows) were gathered and adjacent windows were grouped and considered as insertion hotspots.

1.5. Nomination of dispensable sequences

Each dispensable sequence was assigned a symbol comprised of five letters followed by an eight-digit number (for example, "OsIPC01020013", "OsIPU00011522" and "OsJPC04060073"). Each *indica* dispensable sequence that was assigned a genomic position relative to the Nipponbare reference genome was prefixed with "OsIPC" (Os: *Oryza sativa*, I: *indica*, P: pan-genome, C: contig). The first two digits represented the chromosome identifier while the third and fourth digits indicated the chromosome location of this contig measured in Mb. The last four digits indicated the sequential order of a sequence along a chromosome region in ascending order. Each *indica* dispensable sequence that couldn't be assigned a genomic position relative to the Nipponbare reference genome was prefixed with "OSIPU" followed by randomly assigned eight digit numbers. The same rule was applied to the *japonica* dispensable sequences except that the third letter "J" was used to represent "*japonica*".

References

1. Marcais G, Kingsford C: **A fast, lock-free approach for efficient parallel counting of occurrences of k-mers.** *Bioinformatics* 2011, **27:**764-770.
2. Kelley DR, Schatz MC, Salzberg SL: **Quake: quality-aware detection and correction of sequencing errors.** *Genome Biol* 2010, **11:**R116.
3. Salamov AA, Solovyev VV: **Ab initio Gene Finding in Drosophila Genomic DNA.** *Genome Res* 2000, **10:**516-522.
4. Stanke M, Schoffmann O, Morgenstern B, Waack S: **Gene prediction in eukaryotes with a generalized hidden Markov model that uses hints from external sources.** *BMC Bioinformatics* 2006, **7:**62.
5. Altschul SF, Gish W, Miller W, Myers EW, Lipman DJ: **Basic Local Alignment Search Tool.** *J Mol Biol* 1990, **215:**403-410.
6. Birney E, Clamp M, Durbin R: **GeneWise and Genomewise.** *Genome Res* 2004, **14:**988-995.
