# Supplementary material for: Exploring the rice dispensable genome using a metagenome-like assembly strategy
Source: Genome Biol. 2015 Sep 7;16:187. doi: 10.1186/s13059-015-0757-3 (PMC4583175; doi:10.1186/s13059-015-0757-3)
Supplement: Additional file 18: Table S16. — Functional enrichment analysis of 6302 reference genes involved in the formation of non-reference sequences through exon/intron shuffling. The hmm accession for all the 6302 reference genes was extracted, and the number of genes with a specific hmm accession involved in the formation of non-reference sequences through exon/intron shuffling were compared with the whole genome level to find enriched accessions. In total, 3581 of these 6302 genes and 33,581 genes of the whole genome were annotated by Pfam. (DOC 40 kb) [file 13059_2015_757_MOESM18_ESM.doc]

**Additional file 18: Table S16. Functional enrichment analysis of 6302 reference genes involved in the formation of non-reference sequences through exon/intron shuffling.**

The hmm accesion of all the 6302 reference genes were extracted, and the number of genes with specific hmm accession involved in the formation of non-reference sequences through exon/intron shuffling were compared with the whole genome level to find enriched accessions. In total, 3581 of these 6302 genes and 33581 genes of the whole genome were annotated by Pfam.

| **Hmm accession** | **Hmm name** | **P-value** | **Observed genes** | **Whole**  **genome** |
| --- | --- | --- | --- | --- |
| **PF00075** | RnaseH | 6.50E-29 | 225 | 861 |
| **PF00078** | RVT_1 | 2.52E-114 | 784 | 2874 |
| **PF00098** | zf-CCHC | 4.73E-40 | 455 | 2104 |
| **PF00385** | Chromo | 2.88E-11 | 177 | 943 |
| **PF00665** | rve | 2.72E-137 | 922 | 3385 |
| **PF01419** | Jacalin | 5.40E-05 | 14 | 31 |
| **PF02992** | Transposase_21 | 2.13E-19 | 211 | 960 |
| **PF03578** | HGWP | 3.24E-05 | 48 | 225 |
| **PF03732** | Retrotrans_gag | 5.06E-122 | 817 | 2962 |
| **PF04195** | Transposase_28 | 3.60E-15 | 198 | 977 |
| **PF05970** | DUF889 | 4.19E-05 | 24 | 80 |
| **PF07197** | DUF1409 | 2.90E-42 | 142 | 280 |
| **PF07727** | RVT_2 | 1.39E-59 | 365 | 1205 |
| **PF08284** | RVP_2 | 1.64E-32 | 266 | 1045 |
| **PF10536** | PMD | 7.89E-27 | 185 | 661 |
